# Quantum-Chemical Insights into the Self-Assembly of Carbon-Based Supramolecular Complexes

**DOI:** 10.3390/molecules23010118

**Published:** 2018-01-07

**Authors:** Joaquín Calbo, Juan Carlos Sancho-García, Enrique Ortí, Juan Aragó

**Affiliations:** 1 Institute of Molecular Science, University of Valencia, 46980 Paterna (Valencia), Spain; joaquin.calbo@uv.es (J.C.); enrique.orti@uv.es (E.O.); 2 Department of Physical Chemistry, University of Alicante, E-03080 Alicante, Spain; jc.sancho@ua.es

**Keywords:** noncovalent interactions, carbon-based supramolecular assemblies, quantum chemistry

## Abstract

Understanding how molecular systems self-assemble to form well-organized superstructures governed by noncovalent interactions is essential in the field of supramolecular chemistry. In the nanoscience context, the self-assembly of different carbon-based nanoforms (fullerenes, carbon nanotubes and graphene) with, in general, electron-donor molecular systems, has received increasing attention as a means of generating potential candidates for technological applications. In these carbon-based systems, a deep characterization of the supramolecular organization is crucial to establish an intimate relation between supramolecular structure and functionality. Detailed structural information on the self-assembly of these carbon-based nanoforms is however not always accessible from experimental techniques. In this regard, quantum chemistry has demonstrated to be key to gain a deep insight into the supramolecular organization of molecular systems of high interest. In this review, we intend to highlight the fundamental role that quantum-chemical calculations can play to understand the supramolecular self-assembly of carbon-based nanoforms through a limited selection of supramolecular assemblies involving fullerene, fullerene fragments, nanotubes and graphene with several electron-rich π-conjugated systems.

## 1. Introduction

**Figure 1:**
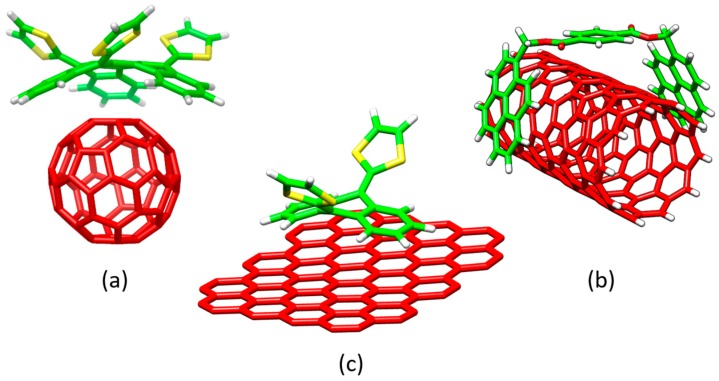
Selected examples of supramolecular assemblies involving fullerene (**a**), nanotubes (**b**) and graphene (**c**) with different π-conjugated electron-donors. The carbon atoms of the electron-acceptor carbon-based systems have been highlighted in red whereas the carbon atoms of the electron-donor systems are colored in green. Sulfur, oxygen and hydrogen atoms in the electron-donor systems are colored in yellow, red and white, respectively.

The self-assembly of molecular entities interacting by weak intermolecular forces to form well-organized and dynamic chemical systems is the heart of the supramolecular chemistry discipline. An optimal molecular assembly is known to be crucial for the generation of complex superstructures (e.g., DNA double-helix or enzymes) with a well-defined functionality. In the last decade, supramolecular chemistry has attracted a great deal of attention, and a tremendous diversity of assembled chemical systems (such as discrete host-guest systems [1,2], metallocages [3,4,5,6,7], supramolecular polymers [8,9,10], covalent-organic frameworks [11], etc.) have been successfully obtained. These supramolecular assemblies present outstanding structural and electronic properties for potential applications in fields ranging from photocatalysis and drug-delivery to photovoltaic and light-emitting devices. In the context of nanoscience, different electron-acceptor carbon-based nanoforms, namely fullerenes, carbon nanotubes, and graphene, have been noncovalently functionalized with electron-donor systems to form discrete donor–acceptor (D–A) supramolecular complexes [2]. For instance, fullerene (C_60_) with its spherical shape has been effectively hosted by different electron-donor curved π-conjugated compounds in a supramolecular approach, where π–π interactions are maximized owing to the structural complementary. The close relatives of C_60_, i.e., carbon nanotubes and graphene, have been also recognized supramolecularly by π-conjugated systems of different nature [12,13,14,15]. Figure 1 displays a few selected examples of supramolecular systems based on carbon electron-acceptor hosts interacting with different electron-donor guests. These carbon-based D–A supramolecular examples have been of great relevance to gain a deep understanding into the fundamental and omnipresent photoinduced electron-transfer process, which is in turn required for organic photovoltaic applications [16,17,18,19]. Carbon-based nanomaterials have also received a great deal of attention due to their potential in medical applications [20,21,22,23,24].

In general, most supramolecular complexes are experimentally characterized in solution, by means of spectroscopic techniques (UV–Vis absorption, ^1^H-NMR, etc.), where an atomically-detailed supramolecular structure is difficult to be obtained. In this context, quantum-chemical techniques can offer an atomically-precise characterization of the supramolecular organization, as well as the nature of the noncovalent interactions (NCIs) responsible for the self-assembly [25,26]. However, the appropriate description of these supramolecular assemblies requires overcoming two challenging problems. First, an accurate treatment of the noncovalent interactions involved in the supramolecular organization is mandatory. Yet, NCIs arise from long-range instantaneous and correlated fluctuations of the electron charge density and, therefore, only quantum-chemical methods able to successfully capture the electron-correlation phenomenon can be employed. In particular, coupled-cluster theory with single, double, and perturbatively-connected triple excitations [CCSD(T)] in combination with large basis sets has become the “gold-standard” method to accurately deal with these weak but important forces in supramolecular systems [27,28,29]. Nevertheless, and here is where the second problem comes out, the use of the CCSD(T) method is restricted to small- or medium-size molecular systems due to its unfavorable computational scaling.

Density functional theory (DFT) is indisputably the most widely used theory for electronic structure calculations in quantum chemistry and condensed matter physics, owing to its reasonable balance between accuracy and computational cost. Unfortunately, the most common density functionals (DFs) available in the literature (local or (semi)local density correlation functionals) are unable to capture the long-range electron correlation phenomenon responsible for NCIs [30,31,32]. In the last decade, this problem has been central in the field of quantum chemistry, and different strategies have been successfully developed [33,34,35,36,37,38,39,40,41,42,43,44]. Among the different approximations proposed, the DFT-D3 approach (developed by Grimme et al. [40]) is likely to be the most popular and extended manner to theoretically treat molecular systems governed by NCIs. This method has demonstrated to provide accurate binding energies in a wide variety of supramolecular complexes of different molecular size with almost no additional computational cost [26,45]. 

The current success in the development of feasible quantum-chemical methods able to accurately describe NCIs at a reasonable trade-off between accuracy and computational cost has paved the way for routine theoretical calculations on relatively large-size supramolecular complexes of wide interest. In this respect, quantum-chemical techniques must be considered nowadays as a powerful tool for characterizing the self-organization in a wide variety of weakly-bound supramolecular complexes. Additionally, the use of these methods is of particular interest in those cases where a detailed experimental characterization is either scarce or difficult to obtain. 

In this review, we demonstrate the potential that quantum-chemical calculations (namely, DFT-D3 calculations) can offer to obtain an in-depth knowledge at the molecular scale of the supramolecular self-assembly between carbon-based electron-acceptor nanoforms (fullerene derivatives, carbon nanotubes and graphene) with different electron-rich π-conjugated motifs. To illustrate this, we have selected a limited number of examples studied by us, and in close collaboration with experimental groups, where quantum-chemical calculations have been decisive to gain a deep understanding of the supramolecular self-organization. This review is mainly aimed to the experimental community and, therefore, the technical details about the different quantum-chemical models have been maintained to be minimum (only a brief and general discussion is given in Section 2). As mentioned above, the examples presented herein have been chosen primarily to highlight the potential of quantum-chemical methods in the context of supramolecular chemistry and, therefore, this review does not intend to be a comprehensive revision of the large number of carbon-based donor–acceptor supramolecular complexes that can be found in the literature.

## 2. Methodology

In this review, we present theoretical examples mostly calculated within the DFT framework with standard density functionals coupled to the Grimme’s dispersion correction (i.e., the DFT-D approximation or in its most modern version DFT-D3 approach). We will begin with a brief description of the density functionals and the semiempirical methods used along the text. Subsequently, we will provide a nutshell overview of the Grimme’s dispersion-corrected approximation. More detailed theoretical reviews about the quantum-chemical methodologies capable of accurately describing noncovalent interactions can be found in References [25,26,28,29,46,47]. Finally, the magnitudes and technical details employed along the review will be briefly explained.

### 2.1. Density Functionals and Semiempirical Methods

A general expression for the exchange-correlation (*xc*) density functionals, *E_xc_*[*ρ*], used in this work can be written as:

(1) $$E_{xc}^{}[\rho ]=w_{HF}E_{x}^{HF}+(1-w_{HF})E_{x}^{}[\rho ]+E_{c}^{}[\rho ],$$

where $E_{x}^{}[\rho ]$ and $E_{c}^{}[\rho ]$ correspond to the generalized gradient approximation (GGA) exchange and correlation energy terms, respectively. The $E_{x}^{HF}$ is the Hartree–Fock (HF) like exchange term. The exchange terms are weighted by the scaling parameter w*_HF_*. Table 1 collects a detailed description for the composition of all the exchange-correlation functionals used in this review.

Semiempirical methods have been widely used for applications in large-size molecular systems. However, they suffer from the same problem as most density functionals being unable to capture long-range electron correlation effects (dispersion forces) and, thus, are not recommended for supramolecular chemistry problems. In the last years, much efforts have been devoted to develop semiempirical methods for general applications including the description of NCIs [58]. Among the different modern semiempirical methods, the Hartree–Fock-based PM7 [59], the HF-3c [60], and the tight-binding DFT (DFTB) [61,62,63] methods (particularly in its variant known as DFTB3) can be highlighted. The PM7 is based on a modified version of the previous PM6 parameterization that not only removes some errors of the neglect of differential diatomic overlap (NDDO) integral approximation but also significantly reduces the average errors in dealing with organic compounds and solid systems. More importantly, PM7 implicitly accounts for weak noncovalent interactions, which allows its use in supramolecular complexes of increasing size. On the other hand, the DFTB3 method is a simplified version of a standard density functional to which dispersion corrected terms as D3 have been successfully coupled. DFTB3-D3 has demonstrated to be a powerful tool to model large supramolecular systems owing to the reasonable balance between accuracy and computational cost. Very recently, Grimme et al. have proposed a novel semiempirical method (GFN-xTB) based on the tight-binding philosophy of DFTB that seems to be very promising [64]. Nevertheless, in this review, we will show results only obtained at the PM7 level for exploratory purposes.

### 2.2. Dispersion Corrections

As mentioned before, the most popular low-cost approach to deal with dispersion interactions within the DFT framework is the Grimme’s correction [40,56,65]. This protocol consists in an atom-pairwise correction to the standard Kohn–Sham density functional to describe the total energy as:

(2) $$E_{\mathrm{DFT}-D3}=E_{xc}^{}[\rho ]+E_{\mathrm{disp}}^{D3},$$

where *E_xc_*[*ρ*] is the self-consistent Kohn–Sham energy (Equation (1)) as obtained from the chosen density functional, and $E_{\mathrm{disp}}^{D3}$ is the dispersion correction term. The dispersion correction term is based on the classical definition of long distance forces [40,56,65] and is directly proportional to the $C_{n,AB}/{\left(R_{AB}\right)}^{n}$ relationship, where $C_{n,AB}$ is a parameterized *n*th-order dispersion coefficient for atom pair *AB*, and ${\left(R_{AB}\right)}^{n}$ is the intermolecular distance between both atoms (*n* can take the values 6, 8, 10, etc.).

In the D3 version of Grimme’s dispersion correction, the dispersion interaction is computed according to the following pairwise energy expression:

(3) $$E_{\mathrm{disp}}^{D3}=-\sum_{n=6,8}s_{n}\sum_{A}^{N_{at}-1}\sum_{B=A+1}^{N_{at}}\frac{C_{n,AB}}{{\left(R_{AB}\right)}^{n}}f_{\mathrm{damp}}(R_{AB})$$

where *s_n_* are customary parameters fitted for individual density functionals. Unlike the former D2 version of Grimme’s correction [56], the dispersion $C_{n,AB}$ coefficients are geometry dependent as they are adjusted on the basis of local geometry (coordination number) around atoms *A* and *B*. $f_{\mathrm{damp}}(R_{AB})$ is a damping function that depends on the distance $R_{AB}$, and may adopt several forms to attenuate the dispersion correction term in the short range [66]. 

The D3 scheme can be further corrected by accounting for the influence of three-body terms (i.e., the energy contributions for all triple atom combinations *A*, *B* and *C*) by means of the Axilrod−Teller−Muto expression [67,68]:

(4) $$E^{ABC}=\overset{N_{at}}{\underset{A<B<C}{\sum }}C_{9,ABC}\frac{\left(3\cos\theta_{a}\cos\theta_{b}\cos\theta_{c}+1\right)}{{\left(R_{AB}R_{BC}R_{AC}\right)}^{3}}\times f_{n}\left(R_{AB},R_{BC},R_{AC}\right),$$

where $\theta_{i}$ are the internal angles of the triangle formed by the interatomic distances $\left(R_{AB},R_{BC},R_{AC}\right)$, $f_{n}\left(R_{AB},R_{BC},R_{AC}\right)$ is a damping function, and $C_{9,ABC}$ is the corresponding coefficient approximated by:

(5) $$C_{9,ABC}\approx -\sqrt{C_{6,AB}C_{6,BC}C_{6,AC}}$$

In addition to the DFT-D3 approach, there is another general and seamless approximation in the field of DFT able to capture dispersion effects in an accurate way. This approach developed by Vydrov and Voorhis is known as the nonlocal approximation (NL) in the quantum chemistry contexts and VV10 in the solid-state physics community [43,44,69]. This approximation takes into account the long-range electron correlation effects (dispersion) by means of an explicit nonlocal density functional correlation kernel, which depends on the electron density at two different sampling points in space, **r** and **r’**. The flexibility of the Vydrov and Voorhis formulation with the incorporation of an adjustable short-range parameter has allowed this NL approximation to be easily merged with a wide variety of standard exchange-correlation density functionals, providing impressive results close to the “chemical accuracy” in small- and medium-size molecular complexes [26,70,71,72,73].

Another interesting dispersion correction is the approximation known as symmetry-adapted perturbation theory (SAPT), which provides a means of directly computing the interaction energy between two molecules without calculating the total energy of the monomers nor the dimer [74]. More importantly, SAPT allows for a decomposition of the interaction energy into physically meaningful components: electrostatic (elst), exchange (exch), induction (ind), and dispersion (disp) terms. In SAPT, the Hamiltonian of the dimer is partitioned into contributions from each monomer and the interaction between them according to:

(6) $$\hat{H}={\hat{F}}_{A}+{\hat{W}}_{A}+{\hat{F}}_{B}+{\hat{W}}_{B}+\hat{V}$$

where the Hamiltonian ($\hat{H}$) is written as a sum of the monomer Fock operators, $\hat{F}$, the fluctuation potential of each monomer, $\hat{W}$, and the interaction potential, $\hat{V}$. The monomer Fock operators, ${\hat{F}}_{A}+{\hat{F}}_{B}$, are treated as the zeroth-order Hamiltonian, and the interaction energy is evaluated through a perturbative expansion of $\hat{V}$, ${\hat{W}}_{A}$ and ${\hat{W}}_{B}$.

Several truncations of the closed-shell SAPT expansion are available. The simplest truncation of SAPT is denoted as SAPT0 [74,75], and is defined by:

(7) $$E_{\mathrm{SAPT}0}=E_{\mathrm{elst}}^{\left(10\right)}+E_{\mathrm{exch}}^{\left(10\right)}+E_{\mathrm{ind},\mathrm{resp}}^{\left(20\right)}+E_{\mathrm{exch}-\mathrm{ind},\mathrm{resp}}^{\left(20\right)}+E_{\mathrm{disp}}^{\left(20\right)}+E_{\mathrm{exch}-\mathrm{disp}}^{\left(20\right)}+\delta_{HF}^{\left(2\right)}$$

where *v* and *w* in *E*^(*vw*)^ refer to the order of *V* and *W_A_* + *W_B_*, respectively, the subscript respectively indicates that orbital relaxation effects are included, and the $\delta_{HF}^{\left(2\right)}$ term takes into account higher-order induction effects.

### 2.3. Binding and Interaction Energies

Along the review, we will use two energy magnitudes for the discussion of the results: the interaction energy (*E*_int_) and the binding energy (*E*_bind_). The former (*E*_int_) is defined as the energy difference between a dimer complex (D) and the individual monomers (M1 and M2) at the geometry of the complex:

(8) $$E_{\mathrm{int}}=E_{D}^{D}-E_{M1}^{D}-E_{M2}^{D}$$

where $E_{X}^{Y}$ is the energy of fragment X at the geometry of Y. The binding (or association) energy (*E*_bind_) is calculated taking into account the relaxation of the separate monomers and, therefore, considering the deformation energy required to transform the isolated monomers from their minimum-energy geometries to the geometry acquired in the assembly:

(9) $$E_{\mathrm{bind}}=E_{\mathrm{def}}+E_{\mathrm{int}},$$

where:

(10) $$E_{\mathrm{def}}=(E_{M1}^{D}-E_{M1}^{M1})+(E_{M2}^{D}-E_{M2}^{M2}).$$

In those examples of large molecular size where small basis sets should be employed to evaluate interaction energies, the basis set superposition error (BSSE) will be corrected according to the counterpoise (CP) method developed by Boys and Bernardi [76]. Basis sets more extended than those of double-ζ quality are expected to present relatively small BSSEs in the interaction energy of supramolecular complexes [77].

### 2.4. Limitations of the Quantum-Chemical Methods

The description of supramolecular complexes at quantum-chemical level is living a flourishing period due to the great efforts made in the last decade [25,26,28,29,46,47]. Nevertheless, all quantum chemistry methods present a balance between accuracy and computational cost (the higher the accuracy, the larger the computational cost). In general, this balance determines the practical use of a particular quantum chemistry method. DFT methods coupled to the Grimme’s dispersion term (DFT-D3) have turned out to be the most practical manner to treat supramolecular complexes [26]. Their computational cost limitation comes mainly from the density functional since the dispersion correction has almost no computational cost [40]. Additionally, the accuracy of the DFT-D3 methods has reached the chemical accuracy (1 kcal/mol) in small- and medium-size supramolecular systems of wide interest [78]. A reasonable trade-off between accuracy and computational cost is also found in large-size systems at the DFT-D3 level [79]. Therefore, we strongly recommend DFT-D3 models for geometry optimizations and calculations of interaction energies in supramolecular complexes below 150 atoms. For larger systems and exploratory purposes, an alternative is the use of semiempirical methods as those briefly described above (PM7, HF-3c, DFTB-D3 and GFN-xTB). Although they are less precise methods, they are very efficient in terms of computational cost and allow extracting valuable theoretical insights, especially by comparing within a family of similar compounds.

## 3. Results

In this section, we present a selection of examples based on different supramolecular assemblies of carbon nanoforms to highlight how relevant structural and energy information can be easily drawn from quantum-chemical calculations to assist in the challenging characterization of large supramolecular assemblies of different nature, and guide in the rationalization of the experimental information available.

### 3.1. Fullerene-Based Donor–Acceptor Supramolecular Assemblies

In the last decade, the quest of molecular receptors capable of effectively hosting fullerenes has attracted a great deal of attention. For an optimal supramolecular interaction with fullerenes, molecular receptors should be designed to incorporate extended π-conjugated surfaces able to maximize the number of π–π interactions [80] with the fullerene derivatives. This requirement has been successfully achieved in molecular curved π-conjugated systems by exploiting the complementarity of the concave shape of the receptor with the convex surface of the fullerene (Figure 1). Nevertheless, not only curved receptors are able to efficiently interact with fullerenes but also planar receptors such as porphyrins have demonstrated to strongly bind C_60_ buckyballs. In this section, we show a few examples of fullerene-based supramolecular complexes with curved and planar π-conjugated receptors where quantum-chemical calculations have played an important role in characterizing their supramolecular self-organization.

#### 3.1.1. Concave extended-Tetrathiafulvalene (exTTF) Derivatives for the Supramolecular Recognition of Fullerenes

**Figure 2:**
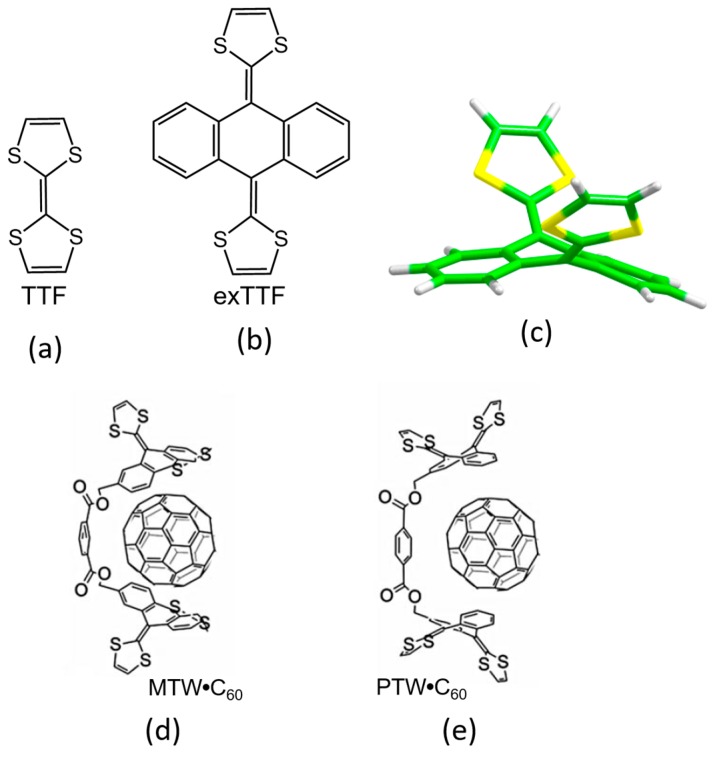
Chemical structure of TTF (**a**) and exTTF (**b**). The curved shape of exTTF is also represented (**c**). Sulfur, carbon and hydrogen atoms are colored in yellow, green and white, respectively. Chemical structures of the supramolecular complexes MTW**•**C_60_ (**d**) and PTW**•**C_60_ (**e**) are also shown.

The 2-[9-(1,3-dithiol-2-ylidene)anthracen-10(9H)-ylidene]-1,3-dithiole compound, also known as exTTF (Figure 2), has aroused great interest in the field of organic electronics due to its resemblance with the popular tetrathiafulvalene (TTF) [81]. Like TTF, the exTTF compound presents a good electron-donor character, and has been combined in numerous donor–acceptor dyads [82] to gain insight into the paramount photoinduced electron-transfer process. Another remarkable feature of exTTF in the context of supramolecular chemistry is its π-conjugated curved (concave) structure. In fact, its particular structure and electron-donor character makes the exTTF system very attractive as a potential supramolecular receptor for fullerenes. Initially, DFT calculations predicted binding energies up to 7.00 kcal/mol between a single unit of exTTF and C_60_. Unfortunately, no experimental evidence of association in solution was found in either UV–Vis or NMR titrations [2]. This apparently negative outcome encouraged the quest of novel chemical entities that incorporate the exTTF moiety for an enhanced C_60_ recognition. In this line, Pérez et al. synthesized a bivalent tweezer with two exTTF units (MTW in Figure 2) able to act as a host, and effectively interact with C_60_ fullerene as a guest owing to the concave–convex structural complementary that maximizes the number of π–π contacts [83]. Subsequently, the supramolecular organization and the photophysical properties of the corresponding discrete donor–acceptor MTW**•**C_60_ and its PTW**•**C_60_ homologue were studied by theoretical calculations and spectroscopic techniques [16].

**Figure 3:**
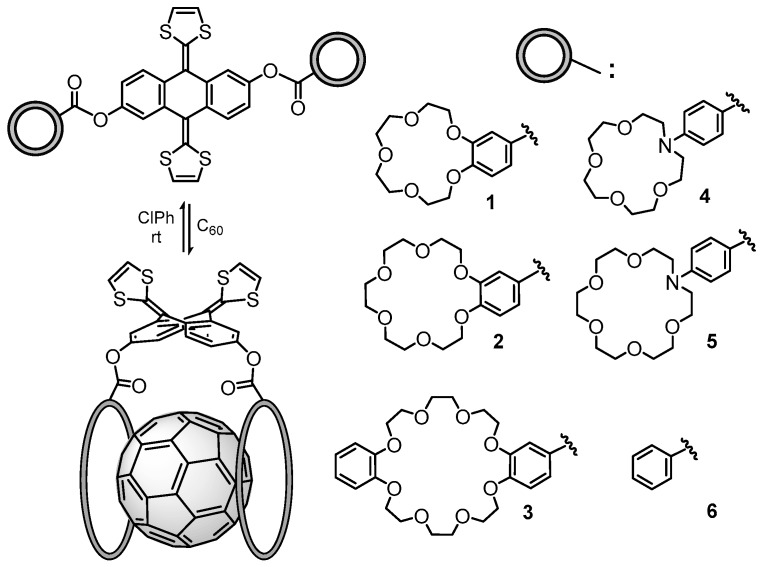
Complexes obtained from exTTF-based **1**–**6** and C_60_. Reproduced from Reference [84] with permission from the Royal Society of Chemistry.

Recently, a series of exTTF derivatives substituted with crown ethers of increasing size specifically designed to host fullerenes were reported (Figure 3) [84]. The aim of this joint experimental‒theoretical study was to unveil the nature of the noncovalent interactions that govern the supramolecular organization between the exTTF-crown ether and C_60_. Experimentally, absorption titrations were performed to confirm the supramolecular complexation between the exTTF derivatives and C_60_. In these experiments, the exTTF absorption bands (350 and 450 nm) gradually decreased upon addition of C_60_. The absorption bands associated with C_60_ were also detected (namely, a strong absorption band at wavelengths <350 nm, a sharp band at 407 nm, and a broad absorption between 470 and 650 nm). Finally, a charge-transfer feature that peaked at longer wavelengths (475 and 485 nm in PhCl and PhCN, respectively) emerged. The spectroscopic data were employed to estimate the association constants for the exTTF molecular tweezers **1**–**6** towards C_60_ in PhCl at 298 K (log *K*_a_ = 4.8 ± 0.9, 6.7 ± 0.2, 6.9 ± 0.2, 3.8 ± 0.6, 5.1 ± 0.1 and 3.3 ± 0.4 M^−1^ for **1•**C_60_, **2•**C_60_, **3•**C_60_, **4•**C_60_, **5•**C_60_ and **6•**C_60_, respectively).

**Figure 4:**
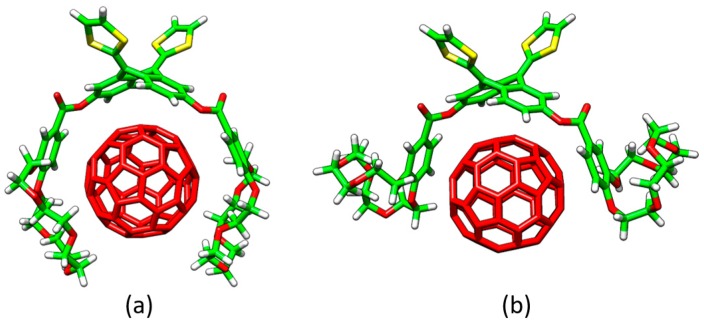
Minimum-energy embraced (**a**) and non-embraced (**b**) conformations calculated at the PM7 level for the **2•**C_60_ complex. The electron-acceptor C_60_ is colored in red whereas the sulfur, carbon, oxygen and hydrogen atoms in the electron-donor system are colored in yellow, green, red and white, respectively.

The different conformations that **1**–**6** may adopt to interact with C_60_ were initially optimized by using semiempirical PM7 calculations, which are recommended for exploratory purposes owing to its favorable computational cost. Figure 4a displays the minimum-energy geometries computed for **2•**C_60_ as a representative example. In all complexes formed by **1**–**6** and C_60_, the fullerene ball interacts with the anthracene concave region of exTTF and, at the same time, the crown ether-based arms embrace C60 in a pinzer-like shape (Figure 4a).

Host–guest arrangements, where the crown ethers are not directly interacting with C_60_, were also optimized for **1•**C_60_, **2•**C_60_ and **3•**C_60_ to evaluate the stabilization owing to the embracing motion (see Figure 4b for **2•**C_60_). For **2•**C_60_, PM7 predicted binding energies of −72.43 and −51.20 kcal/mol for the embraced and non-embraced conformations, respectively (Figure 4). Similar results were obtained for the other complexes calculated (**1•**C_60_ and **3•**C_60_) [84]. PM7 calculations were therefore key to estimate quantitatively the energy stabilization of the embracing motion promoted by the exTTF-based hosts for the recognition of fullerene C_60_.

**Figure 5:**
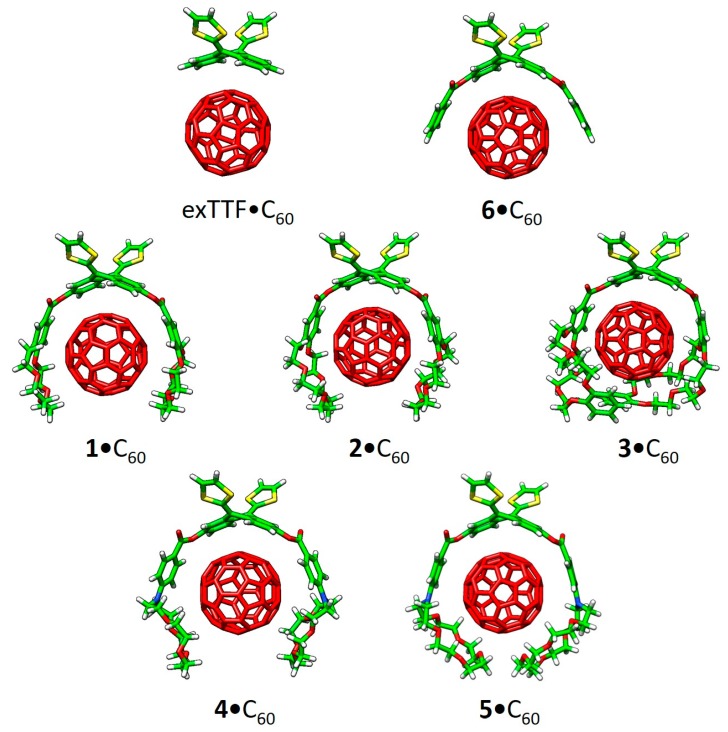
B97-D/cc-pVDZ minimum-energy geometries calculated for the exTTF•C_60_ and **1**–**6•**C_60_ complexes. The electron-acceptor C_60_ is colored in red whereas the sulfur, carbon, oxygen and hydrogen atoms in the electron-donor system are colored in yellow, green, red and white, respectively.

The more stable embraced conformations were then optimized using the dispersion-corrected B97-D functional in combination with the cc-pVDZ basis set (Figure 5). The exTTF**•**C_60_ complex was also computed as a reference. Note that the B97-D density functional presents an outstanding trade-off between accuracy and computational cost and, thus, is highly recommended for exploratory and optimization purposes [56]. The optimized structures showed intermolecular contacts of different nature along the host–guest interacting region. Table 2 collects the shortest intermolecular contacts determining the stabilization of the complexes between the receptors **1**–**6** and C_60_, as well as the binding energies computed for the resulting supramolecular complexes. To assess the binding energies, single-point energy calculations were carried out on the B97-D/cc-pVDZ-optimized structures using the revPBE0-D3 functional and the more extended triple-ζ cc-pVTZ basis set. Note that triple-ζ basis sets are highly recommended, if the molecular size allows it, to yield reasonable binding energies in weakly-interacting molecular systems with minimal basis set superposition error [77]. A stable exTTF**•**C_60_ complex (*E*_bind_ of −10.24 kcal/mol) was obtained due to π–π interactions between the lateral benzene rings of exTTF and the benzene rings of C_60_ with centroid–centroid distances of 3.42 Å (**a** in Table 2). As exTTF•C_60_ has not been detected experimentally to date, entropic and solvent effects are expected to counterbalance this stabilizing interaction. In the **6•**C_60_ complex, two additional interactions coming from the presence of the benzoates were found: π–π interactions at 3.25 Å between the benzene rings of the benzoate moiety and C_60_ (**b** in Table 2), and *n*–π interactions owing to short O(host)···C(guest) intermolecular distances (3.16 Å, **c** in Table 2). The stabilizing effect of these NCIs was demonstrated by the folding angle of the anthracene skeleton in exTTF, which becomes sharper in going from exTTF•C_60_ (142.5°) to **6**•C_60_ (137.0°). The binding energy for **6**•C_60_ was calculated to be −22.85 kcal/mol, which is more than twice of the binding energy obtained for exTTF•C_60_. The significant stabilization predicted in the **6**•C_60_ formation supported the experimental detection of this complex in solution.

Moving to larger complexes (from **6**•C_60_ to **1**•C_60_, **2**•C_60_, and **3**•C_60_) where crown ethers are inserted in the electron-donor host system, new *n*–π (**d**) and CH···π (**e**) interactions with intermolecular distances of around 2.9 and 2.6 Å emerge to stabilize the resulting supramolecular assembly (Table 2). revPBE0-D3 calculations predicted binding energies going from −39.69 kcal/mol for 1•C_60_, to −44.76 kcal/mol for **2**•C_60_, and to −54.36 kcal/mol for **3**•C_60_. This trend was in line with the increase of the *K*_a_ value obtained experimentally, and was attributed to the increasing size of the crown ethers when going from **1•**C_60_ to **3•**C_60_ (i.e., increasing number of stabilizing *n*–π and CH···π interactions). The crown ether arms wrap C_60_ and lead to more compact complexes, in which the benzene rings of the benzoate moiety are closer (by 0.2 Å) to C_60_ as compared, for example, with **6**•C_60_ (distance **b** in Table 2). This gain in compactness reinforces the stabilizing effect that NCIs between C_60_ and the crown ethers exert on the resulting complex stability.

**Figure 6:**
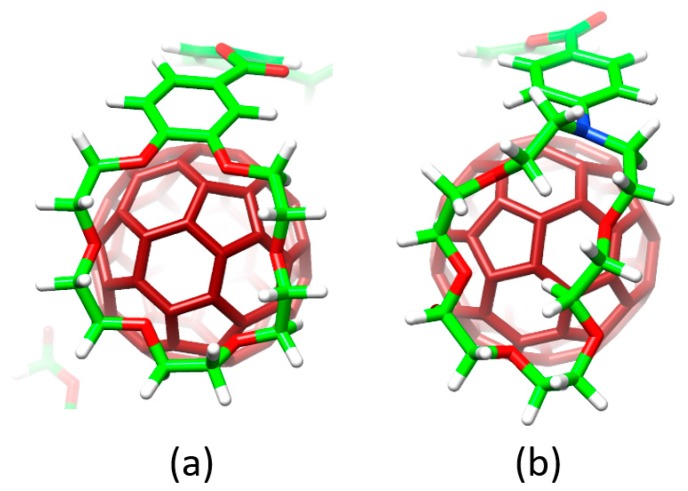
Side view of the B97-D/cc-pVDZ-optimized geometries calculated for complexes **2**•C_60_ (**a**) and **5**•C_60_ (**b**) showing the different spatial arrangement of the crown and aza-crown ethers, respectively, along the C_60_ guest. The electron-acceptor C_60_ is colored in red whereas the sulfur, carbon, oxygen and hydrogen atoms in the electron-donor system are colored in yellow, green, red and white, respectively.

Finally, the effect of the nitrogen atoms on the capability of aza-crown ether derivatives **4** and **5** to host the C_60_ species was analyzed. The insertion of the nitrogen bridge between the benzoate units and the crown ethers confers additional flexibility to the aza-crown ethers in **4**•C_60_ and **5•**C_60_. This flexibility gives rise to structures more folded than those computed for the oxygenated analogues **1•**C_60_ and **2•**C_60_ (Figure 6). Smaller binding energies for **4•**C_60_ and **5•**C_60_ (−36.77 and −43.33 kcal/mol, respectively) were found compared to the oxygenated complexes **1•**C_60_ and **2•**C_60_ (−39.69 and −44.76 kcal/mol, respectively). The lower affinity to C_60_ obtained for the aza-crown ethers is in line with the experimentally determined binding constants, and was associated to an overall weakening of the host–guest interactions caused by the less favorable aza-crown ether arm orientations. 

This example satisfactorily illustrated how quantum-chemical calculations can be used as a very useful tool to rationalize the supramolecular assemblies formed by electron-donor hosts and the C_60_ guest. In particular, calculations pointed out that the ability of the exTTF-based molecular tweezers to bind C_60_ comes from an interplay of different π–π, *n*–π and CH···π interactions, and that the size and nature of the crown ether are key factors for the relative stabilization of the resulting complexes between **1**–**6** and C_60_.

#### 3.1.2. Curved Truxene-Tetrathiafulvalene (truxTTF) Donor as a Supramolecular Partner for Fullerenes

**Figure 7:**
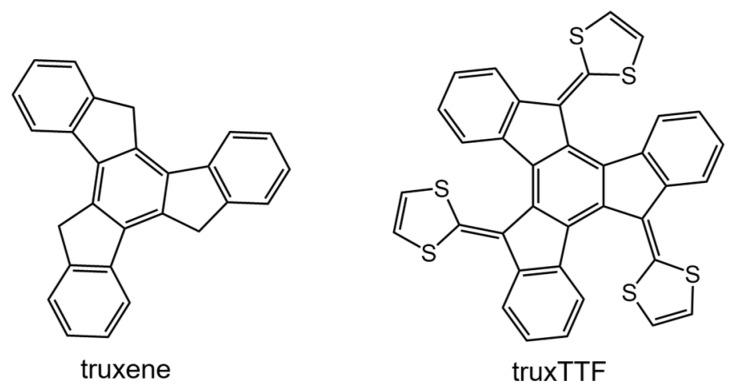
Chemical structure of the truxene and truxTTF compounds.

Among the different carbon-based nanofragments, the truxene structure (Figure 7) has attracted a great deal of interest due to its exceptional solubility, high thermal stability and ease to be chemically modified [85]. Over the last years, and owing to the advances in the synthesis of truxene derivatives, the scope of applications of this attractive heptacyclic polyarene building block, initially limited to synthesis and photoluminescence, has been extended to organic electronics [86]. Particularly interesting is the modification of the truxene core through the incorporation of three dithiole rings to give rise to the electron-donor truxene-TTF (truxTTF) compound (Figure 7) [87]. In analogy to the exTTF derivatives (Section 3.1.1), the truxTTF structure is distorted out of planarity due to short dithiole–benzene contacts, generating a double-concave cavity that fulfills the structural requirements as a host for the supramolecular recognition of fullerene derivatives.

In 2007, Pérez et al. conducted a joint experimental‒theoretical study, and demonstrated that the truxTTF electron-donor was able to effectively host fullerenes due to its concave structure [87]. Experimentally, the supramolecular association between truxTTF and C_60_ was investigated by ^1^H-NMR titrations. The generation of the bimolecular 1:1 supramolecular complex with a binding constant of 1.2 × 10^−3^ M^−1^ (see more experimental details in reference [87]) was confirmed. Additionally, ^1^H-NMR experiments suggested that the supramolecular interaction occurred preferentially on the aromatic truxene surface of truxTTF.

Notwithstanding the experimental evidences about the formation of the donor‒acceptor truxTTF•C_60_ complex, detailed information on the supramolecular organization at atomistic level was not reported so far. To unravel the origin and nature of the interactions between the host and the guest, DFT calculations at the MPWB1K/6-31G** level were performed. The MPWB1K density functional, developed by Truhlar et al., was designed to capture π–π interactions in stacked DNA pairs and amino acid pairs [88]. Note that in 2007, the Grimme’s dispersion-corrected approximation in its most modern version (DFT-D3) was not still developed and this kind of density functionals were a practical solution for the theoretical treatment of weakly-bound molecular systems. Nowadays, the DFT-D3 or DFT-NL approximations would be more recommended for general supramolecular applications [26,69].

**Figure 8:**
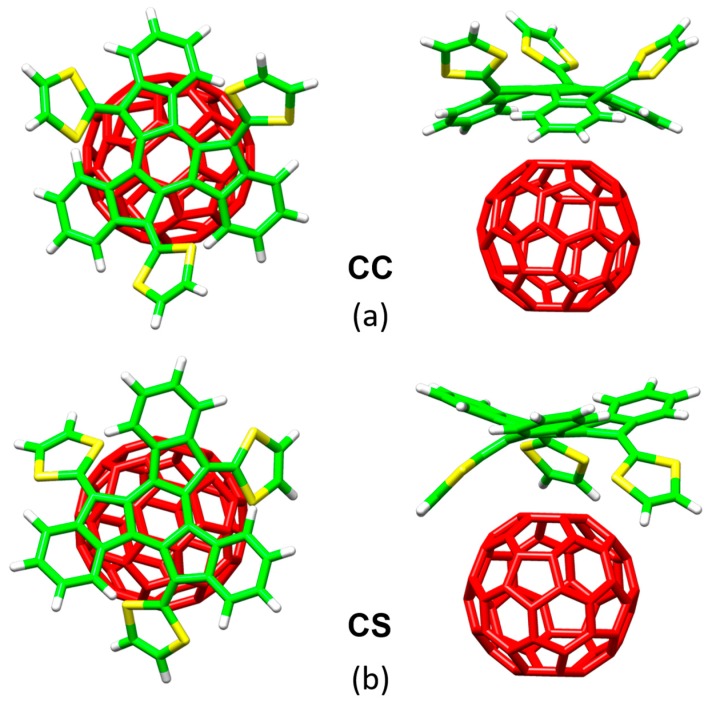
Minimum-energy structures of the truxTTF•C_60_ complex calculated at the MPWB1K/6-31G** level. Top and side view of structures **CC** (**a**) and **CS** (**b**) are given. The electron-acceptor C_60_ is colored in red whereas the sulfur, carbon and hydrogen atoms in the electron-donor system are colored in yellow, green and white, respectively.

Figure 8 displays the minimum-energy geometries calculated at the MPWB1K/6-31G** level for two different structures of the truxTTF•C_60_ complex. In structure **CC**, fullerene C_60_ was placed inside of the aromatic cavity created by the truxene core, stabilized by multiple π–π contacts. This structure clearly exploited the requirement of structural concave–convex complementary to form the host-guest associate. The central benzene ring of the truxene core stacks on one of the benzene rings of C_60_ in a slightly parallel-displaced fashion, in which the benzene rings are twisted by approximately 20° relative to each other and are separated at an average distance of 3.39 Å. This distance turned out to be considerably shorter than the distances reported for the benzene dimer in parallel (3.9 Å) and parallel-displaced (3.6 Å) configurations [89]. In addition to the interactions of the central benzene rings, the peripheral benzene rings of truxTTF exhibit many intermolecular contacts in the 3.6–3.9 Å range with the C_60_ guest that contribute to the stabilization of the complex. Following with the concave–convex complementary concept, the concave cavity formed by the three dithiole rings of the truxTTF electron-donor is also able to bind C_60_ as shown in structure **CS** (Figure 8), with short S···C intermolecular contacts (3.5–3.6 Å). In terms of stability, structure **CC** was calculated to be the most stable with an interaction energy of −8.98 kcal/mol, whereas structure **CS** presented an interaction energy of −2.28 kcal/mol.

In this example, DFT calculations were key to visualize the supramolecular structure of the truxTTF•C_60_ complex, and confirm the experimental evidence about the preferable supramolecular orientation between the electron-donor truxTTF host and the electron-acceptor C_60_ guest. Additionally, this truxTTF•C_60_ supramolecular complex has been of great relevance for the design and understanding of other related supramolecular systems that will be described in Section 3.2.

#### 3.1.3. Metalloporphyrin-C_60_ Associates

**Figure 9:**
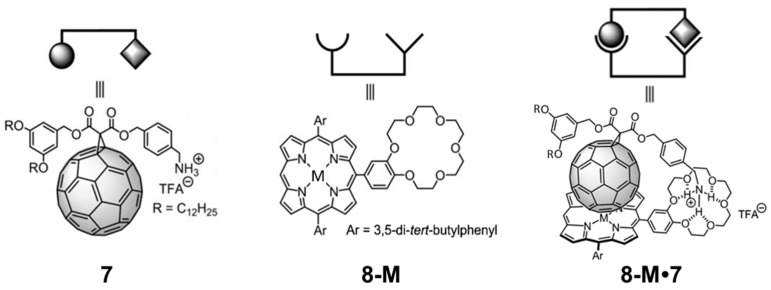
Chemical structure of the methano[60] fullerene guest **7** (**Left**), the metalloporphyrin host **8-M** (**Center**), and the host–guest supramolecular complex **8-M•7** (**Right**). **M** refers to either 2H, Co., Ni, Cu or Zn.

As mentioned above, not only curved electron-donor π-conjugated compounds can act as efficient receptors for fullerene balls. Porphyrins have shown to present an outstanding ability to effectively interact with C_60_ by π–π interactions despite the planar‒convex mismatch [80]. Recently, we thoroughly investigated a series of novel cup-and-ball metalloporphyrin–fullerene conjugates in close collaboration with the experimental groups of Martín and Nierengarten [90]. Our main goal was to shed light onto the nature and strength of the noncovalent interactions governing the supramolecular assembly of fullerene derivatives with metal-substituted porphyrins. Figure 9 displays the chemical structures of the corresponding porphyrin–crown ether conjugates (**8-M**; **M** = 2H, Co., Ni, Cu or Zn) and the methano[60] fullerene derivative **7** [91], as well as the target complexes **8-M**•**7**. Experimentally, the supramolecular complexation was first evidenced through ^1^H-NMR spectroscopy, and the measurement of the binding constant of **8-M**•**7** was performed by monitoring the changes in the UV–Vis absorption spectra. The logarithmic binding constants (log *K*_a_) for porphyrins **8-M** with the methanofullerene derivative **7** at 25 °C in dichloromethane were 5.5, 6.3, 5.9, 6.3 and 6.9 for **8-2H**•**7**, **8-Co**•**7**, **8-Ni**•**7**, **8-Cu**•**7** and **8-Zn**•**7**, respectively.

**Figure 10:**
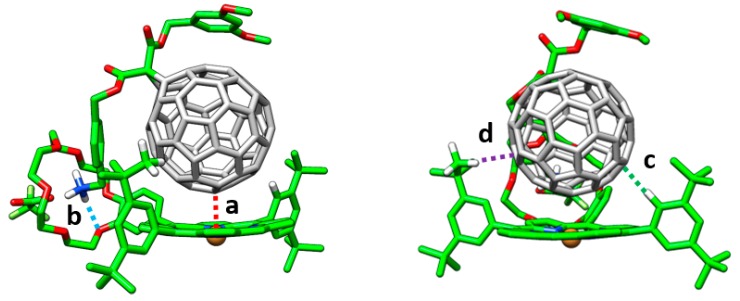
Minimum-energy geometries calculated for the **8-Cu•7** complex at the PM7 level. Side (**Left**) and front (**Right**) views are displayed. The different types of intermolecular contacts are denoted with labels a–d. Only relevant hydrogen atoms are displayed for clarity. Except for C_60_ carbon atoms depicted in grey, the following color code is used: carbon in green, nitrogen in blue, oxygen in red and hydrogen in white.

To better understand the nature of the different interactions governing the assemblies and gain insight into the experimental ordering found for the association constants (*K_a_*), a comprehensive theoretical investigation of these supramolecular complexes was conducted in a multi-level approach. Geometry optimizations were initially performed at the semiempirical PM7 level and showed that, after full geometry relaxation, the ammonium group of the methanofullerene interacts with the crown ether of the porphyrin by H-bond formation (Figure 10). Otherwise, the fullerene ball recognizes the center of the porphyrin system interacting by NCIs. Subsequent DFT reoptimizations at the B97-D/6-31G* level of theory led to the supramolecular parameters summarized in Table 3.

The main interactions determining the supramolecular assembly are represented by the M–C_60_ (a) and NH···O (b) distances provided in Table 3 (Figure 10 for labelling). In addition, CH···π dispersion interactions (c and d) between the *tert*-butyl substituted benzene rings of the porphyrin and the π-cloud of the C_60_ buckyball also contribute to the supramolecular stabilization.

The binding energy for the **8-M**•**7** complexes was estimated at the PBE0-D3/cc-pVTZ level of theory by using the B97-D/6-31G*-optimized geometries (Table 3). Ebind increases from −88.7 in **8-Ni**•**7** to −92.8 kcal/mol in **8-Zn**•**7** due to the more stabilizing M–C_60_ interaction that takes place in moving to electron-richer metal atoms. The stabilization for the non-metalated **8-2H**•**7** complex amounts to −92.4 kcal/mol, and the largest binding energy was computed for **8-Co**•**7** (−93.8 kcal/mol). Theoretical calculations showed that NCIs between the fullerene ball and the phenyl-substituted porphyrin amounts to −22.5 kcal/mol, and the presence of the *tert*-butyl groups at the meta position of the phenyl rings (interaction d) causes an additional stabilization of ~4 kcal/mol (−26.3 kcal/mol in total), in good accord with previous theoretical studies [93]. The ammonium–crown ether NH···O contacts was found to be the main stabilizing driving force, with an interaction energy of −64.9 kcal/mol, which is three times the stabilization of the porphyrin–C_60_ interaction.

**Figure 11:**
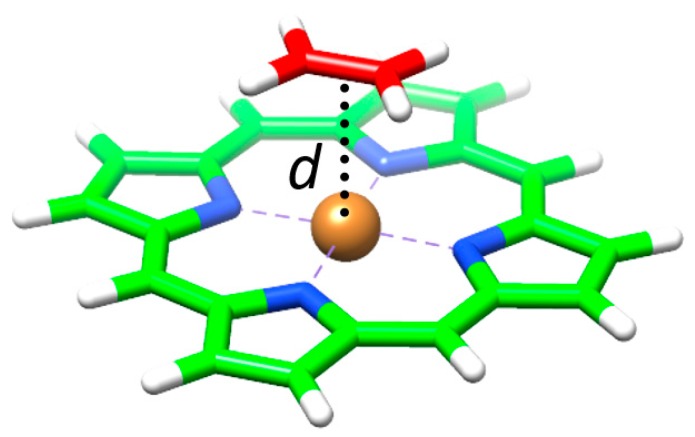
Porphyrin•C_2_H_4_ model (**MP**•C_2_H_4_) used to understand the nature of the interaction between the porphyrin host **8** and the fullerene derivative guest **7**. Except for the ethylene carbon atoms depicted in red, the following color code is used: carbon in green, nitrogen in blue and hydrogen in white.

Taking into account that C_60_ interacts with the porphyrin moiety mainly through one electron-rich [6,6] double bond [94], we computed a simplified model (**MP**•C_2_H_4_), in which the pristine porphyrin (**MP**) interacts with a molecule of ethylene (Figure 11). This reduced model allowed performing more accurate calculations to better understand the relative stabilization of the different assemblies when substituting the metal in the porphyrin. A clear correlation between the calculated binding energy and the metal–ethylene distance (*d*) was found: the shorter the distance along the series **NiP**•C_2_H_4_ (3.18 Å) > **CuP**•C_2_H_4_ (3.00 Å) > **ZnP**•C_2_H_4_ (2.75 Å) > **CoP**•C_2_H_4_ (2.62 Å), the larger the stabilization of the complex (Table 4). The net charge calculated for the metal atom was demonstrated to increase in going from **CoP**•C_2_H_4_ (+0.720e) to **ZnP**•C_2_H_4_ (+1.223e) —Table 4— and, in a first approach, this was related to the stabilizing electrostatic interaction between the porphyrin and the C_60_ guest.

Symmetry-adapted perturbation theory (SAPT) calculations based on the Hartree–Fock wavefunction were performed for **2HP**•C_2_H_4_, **NiP**•C_2_H_4_ and **ZnP**•C_2_H_4_ to decompose the total binding energy into electrostatic, exchange, induction and dispersion energy components (Table 5). A stabilization in the electrostatic term of more than 10 kcal/mol was predicted in passing from **NiP**•C_2_H_4_ to **ZnP**•C_2_H_4_, whereas the exchange interaction was computed positive, and much larger for **ZnP**•C_2_H_4_ than for **2HP**•C_2_H_4_ and **NiP**•C_2_H_4_. The induction term is meant to decay with the distance between the two interacting moieties as *R*^−*n*^, where *n* = 2–4, and thus it was computed to be non-negligible only in the case of the best interacting **ZnP**•C_2_H_4_ (Table 5). Finally, the dispersion energy was predicted to be the largest stabilizing contribution in **2HP**•C_2_H_4_ and **NiP**•C_2_H_4_, and it also largely stabilized **ZnP**•C_2_H_4_ in more than 10 kcal/mol. Theoretical calculations therefore suggest that the energy term that mainly contributes to the stabilization of the **2HP**•C_2_H_4_ assembly is the dispersion component, whereas the electrostatic contribution acquires a major role in the metal-based porphyrin complexes, especially in **ZnP**•C_2_H_4_, for which M–ethylene distances are computed shorter and the metal bears a larger positive charge. 

This supramolecular example clearly highlights the potential of the theoretical calculations to provide not only precise structural information of the self-assembly but also a detailed analysis of the main forces (electrostatic, exchange-repulsion, induction, dispersion, etc.) contributing to the stabilization of supramolecular complexes.

#### 3.1.4. Ditopic Porphyrin•C_60_ complexes

**Figure 12:**
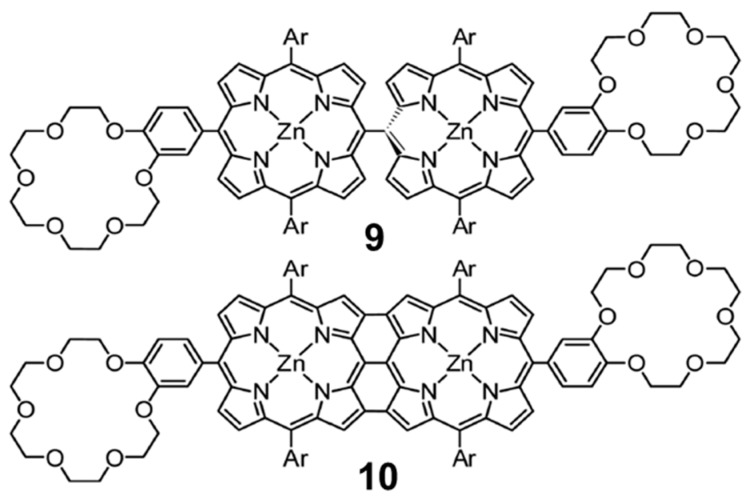
Chemical structure of the ditopic porphyrin-based hosts **9** and **10**.

Whereas the supramolecular chemistry involving porphyrin and fullerene has been extensively explored through the generation of associates involving porphyrin tweezers and cages [95,96] with metal–ligand bonds [97,98], hydrogen bonds [99,100,101], electrostatic interactions [102], mechanical bonds [103,104], or a combination of several of these interactions [105,106], supramolecular arrays involving conjugated multiporphyrin systems are, however, scarce in the literature. With the experienced gained in the work described above on the monotopic metalloporphyrin–fullerene conjugates, we undertook a collaborative study of the supramolecular complexation of the methano[60]fullerene compound **7** shown in Figure 9 by the two analogous ditopic porphyrin receptors, *meso–meso* **9** and tape **10**, displayed in Figure 12. A 1:2 stoichiometry was foreseen for both **9** and **10** when coupled to **7** based on the design of the host molecules, which was further corroborated by Electro-Spray Ionization (ESI) mass spectroscopy experiments. 

**Figure 13:**
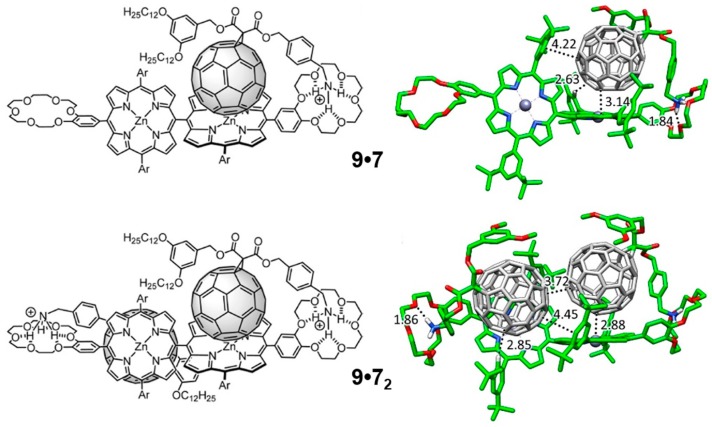
Chemical structure (**Left**) and minimum-energy geometry calculated at the B97-D3/(6-31G**+LANL2DZ) level (**Right**) of the supramolecular assembly of ditopic host **9** with one and two molecules of guest **7**. Except for C_60_ carbon atoms depicted in grey, the following color code is used: carbon in green, nitrogen in blue, oxygen in red and hydrogen in white. Adapted with permission from [90]. Copyright 2014 American Chemical Society.

Theoretical calculations were performed to shed light into the nature and strength of the interactions controlling the different supramolecular association processes, with special attention to the negative cooperative effects experimentally evidenced for these systems (see the original Reference [90] for further details). Minimum-energy geometries were calculated for supramolecular complexes **9**•**7** and **9**•**7_2_** at the B97-D3/(6-31G**+LANL2DZ) level of theory (Figure 13). Similarly to the monotopic porphyrin supramolecular arrangements discussed in the previous Section 3.1.3, compound **7** in **9**•**7** interacts with the crown ether through the positively-charged ammonium group, forming three NH···O(ether) hydrogen-bond interactions in the 1.83–2.00 Å range. Additional short H···C contacts between the peripheral *tert*-butyl-substituted phenyl rings and C_60_ were computed in the range of 2.5–3.2 Å, which add approximately 1 kcal/mol of stabilization per each interaction. Importantly, the vicinal porphyrin, linked to the porphyrin that interacts with **7**, approaches the fullerene fragment and gives rise to additional interactions: short H···C contacts in the 2.7–3.2 Å range and a weak π–π interaction between the peripheral benzene ring and the fullerene.

Moving to the 1:2 stoichiometric complex **9**•**7_2_**, the second molecule of **7** occupies the empty porphyrin surface, and defines similar interactions to those described for **9**•**7**. The minimum-energy geometry showed that the two fullerenes tend to approximate each other in order to stabilize the resulting complex, with close C···C contacts between the two buckyballs of 3.7 Å (Figure 13). This was at the expense of distortions out from orthogonality between the two porphyrin units. The peripheral di-*tert*-butylphenyl groups placed on the vicinal porphyrin moieties plays an active role in the stabilization of the complex with short H···C(C_60_) contacts around 2.8 Å and π–π interactions at 4.4 Å.

**Figure 14:**
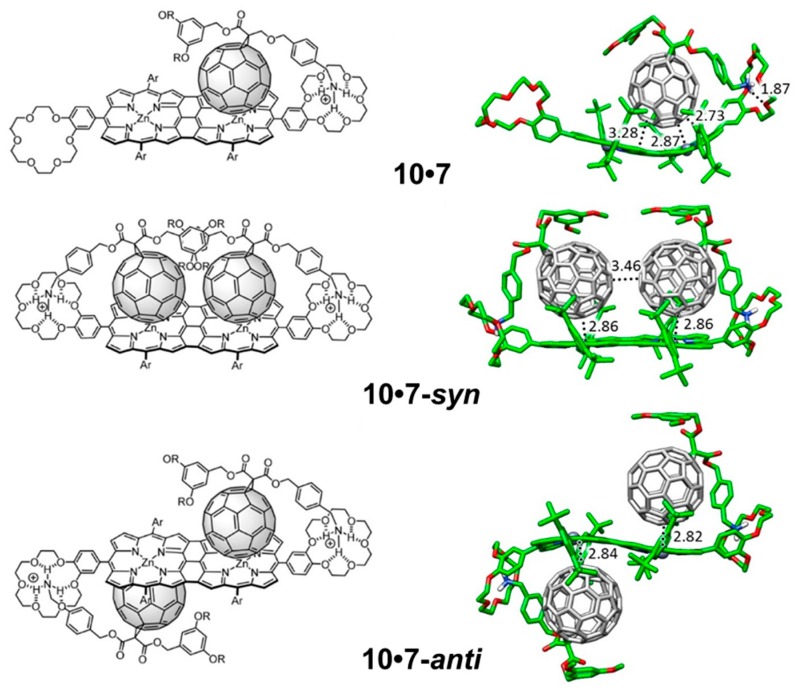
Chemical structure (left) and minimum-energy geometry calculated at the B97-D3/(6-31G**+LANL2DZ) level (right) for the supramolecular assembly of ditopic host **10** with one and two molecules of guest **7**. Except for C_60_ carbon atoms depicted in grey, the following color code is used: carbon in green, nitrogen in blue, oxygen in red and hydrogen in white. Adapted with permission from [90]. Copyright 2014 American Chemical Society.

The supramolecular organization between porphyrin tape **10** and **7** (Figure 14) follows the same pattern as previously described for **9**•**7**. Here, two different possibilities can be designed for the introduction of the second fullerene-based guest **7** into the **10**•**7** complex: the two fullerene balls standing in the same side in a *syn* disposition (**10**•**7_2_-*syn***), or the two balls located in opposite sides with respect to the plane generated by the porphyrin tape dimer in an *anti* disposition (**10**•**7_2_-*anti***) (Figure 14). In the former, an important π–π stabilization originates from the fullerene–fullerene proximity. Experiments demonstrated that the association of the first molecule of **7** in porphyrin hosts **9** and **10** led to a complex where the incorporation of a second equivalent of **7** was more difficult (negative cooperativity). The effective π–π interactions between the buckyballs found for **10**•**7_2_-*syn*** confers an additional stabilization that would reduce the negative cooperativity with respect to **9**•**7_2_-*anti***, as experimentally evidenced for the tape assembly.

Single-point energy B97-D3 calculations were performed on the optimized geometries by using the more extended cc-pVTZ+LANL2DZ basis set to estimate *E*_bind_ for all the supramolecular complexes (Table 6). The association of one molecule of **7** by the *meso–meso* porphyrin dimer **9** led to a large net stabilization of −108.19 kcal/mol, rising especially from the NH···O(ether) contacts and the porphyrin core–C_60_ interaction. Upon insertion of the second molecule of **7**, *E*_bind_ is approximately doubled, reaching a value of −211.05 kcal/mol for **9**•**7_2_**. In the case of **10**, the insertion of the second molecule of **7** in a *syn* disposition is energetically favored due to the π–π interactions between the buckyballs. The theoretical values calculated for *E*_bind_ therefore indicate that the incorporation of the first guest molecule leads to a more stable complex for **9** than for **10**, and suggest that the entrance of the second molecule of **7** is relatively more favored for **10** than for **9** (binding energy differences per unit of **7** between 1:2 and 1:1 complexes of +5.33 and −3.4 kcal/mol for **9** and **10**, respectively; Table 6). These trends were in good agreement with the higher association constant *K*_1_ obtained for *meso–meso* porphyrin **9** (log *K*_1_ = 8.7) compared to tape porphyrin **10** (log *K*_1_ = 6.8), and with the smaller decrease that the association constant experiences for **10** in passing from the 1:1 to the 1:2 stoichiometry (log *K*_2_ = 5.4 for both **9**•**7_2_** and **10**•**7_2_**).

To rationalize the experimental trends in the association constant for both the 1:1 and 1:2 supramolecular complexes, net electronic charges were computed at the B97-D3/(6-31G**+ LANL2DZ) level for **9**•**7** and **10**•**7** using the Natural Population Analysis (NPA) approach. Upon formation of complexes **9**•**7** and **10**•**7**, the electron-donor porphyrin dimer transfers 0.19e and 0.26e to the fullerene-based acceptor, respectively. In **9**•**7**, the porphyrin moiety interacting with the C_60_ ball accumulates a positive charge of +0.16e, whereas the vicinal empty porphyrin bears a residual positive charge of only +0.03e. Moving to **10**•**7**, the C_60_-interacting porphyrin moiety bears a smaller positive charge of +0.11e compared to the empty porphyrin fragment (+0.15e). The efficient π-conjugation between the two porphyrin moieties in tape **10** allowed to explain the charge transfer from one fragment to the other. Theoretical calculations therefore predicted a notable decrease in the electron density for both *meso–meso* and tape porphyrin dimers in the ground state upon complexation of the first acceptor molecule of **7**. The decrease of electronic density disfavors the entrance of the second guest molecule, and contributes to the remarkable change of the association constant (log *K*_a_), from 8.7 to 5.4 in **9**•**1_2_** and from 6.8 to 5.4 in **10**•**7_2_**, when the second molecule of **7** is introduced to form the stoichiometric 1:2 complex. For complex **10**•**7_2_**, the stabilizing interaction between the C_60_ units found for the more stable *syn* disposition partially compensates the negative effect caused by the reduced electronic density, inducing a reduction of the negative cooperativity as evidenced by experiments.

### 3.2. Supramolecular Communication between Buckybowls and the Curved truxTTF Donor

In the last decade, other kinds of carbon nanoforms different to the ubiquitous electron-acceptor C_60_ have gained an increasing attention. Based on the alternation of 5- and 6-member fused rings, fullerene fragments (also known as buckybowls) have aroused the interest of the scientific community as models of their parent buckyballs, nanotube and graphene materials. Buckybowls have the added value of a richer chemistry due to the edges and of their pure synthetic availability with a well-defined molecular structure [107].

#### 3.2.1. The truxTTF•hemifullerene Supramolecular Complex

**Figure 15:**
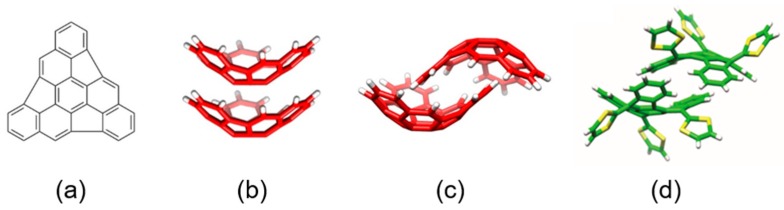
Chemical structure of the hemifullerene C_30_H_12_ buckybowl (**a**); Structure of the dimers formed by C_30_H_12_ (carbon atoms in red) in its trigonal (**b**) and orthorhombic (**c**) crystal polymorphs, respectively; (**d**) Dimers formed by truxTTF in the crystal. For C_30_H_12_, the carbon atoms are depicted in red and hydrogen atoms in white. For truxTTF, the sulfur, carbon and hydrogen atoms are colored in yellow, green and white, respectively.

Hemifullerene (C_30_H_12_) is a curved polycyclic aromatic hydrocarbon compound (buckybowl) that was firstly synthesized in 2004 [108]. In the solid state, two polymorphs were found, each of which exhibits a different packing arrangement, originating from the interaction between the C_30_H_12_ molecules. Figure 15 displays the chemical structure of the hemifullerene C30H12 and the dimeric arrangements found in its two crystal polymorphs. In the trigonal polymorph, bowl-in-bowl stacks were found, an orientation in which π–π interactions are maximized (Figure 15b). In the orthorhombic polymorph, each hemifullerene inserts one of its six-membered rings into the cavity of a neighboring molecule, forming dimers in which both CH···π and π–π interactions play a primary role (Figure 15c). On the other hand, truxTTF, for which the bowl-in-bowl arrangement is prevented by the protruding dithiole rings, presents a dimeric crystallographic arrangement in which one of the aromatic rings of each monomer is placed inside the cavity of the other (Figure 15d) [87].

**Figure 16:**
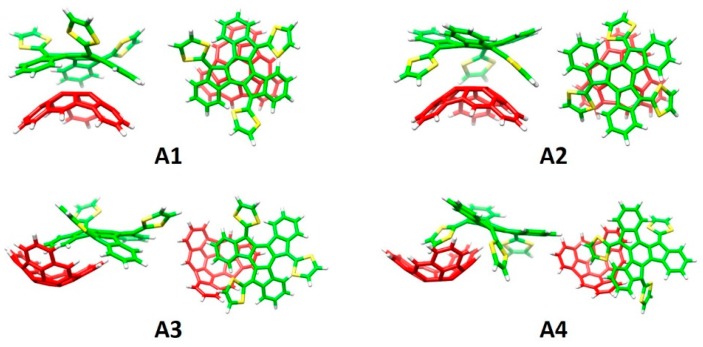
Minimum-energy structures (**A1**–**4**) computed for the truxTTF•C_30_H_12_ heterodimer at the revPBE0-D3/cc-pVTZ level. For C_30_H_12_, the carbon and hydrogen atoms are depicted in red and white, respectively. For truxTTF, the sulfur, carbon and hydrogen atoms are colored in yellow, green and white, respectively.

**Figure 17:**
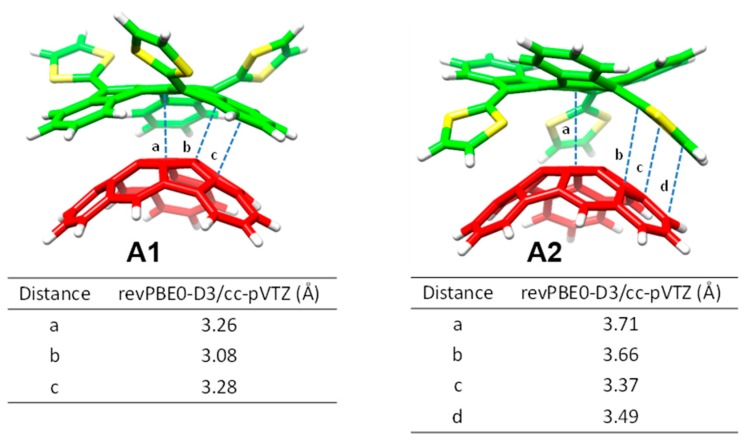

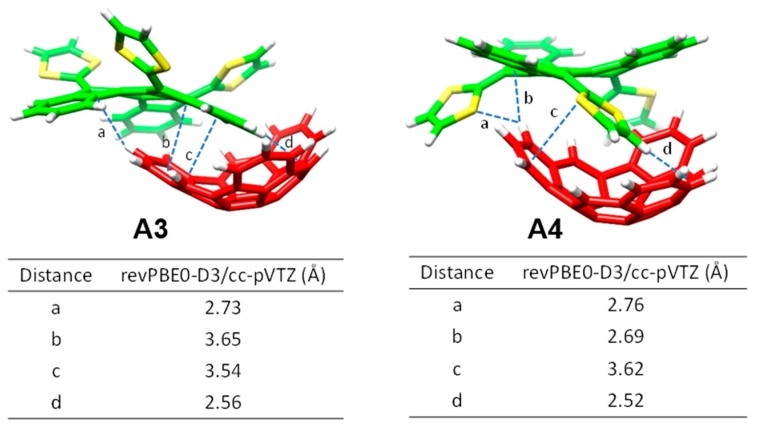
Selected intermolecular distances computed for structures **A1**–**A4** at the revPBE0-D3/cc-pVTZ level. For C_30_H_12_, the carbon and hydrogen atoms are depicted in red and white, respectively. For truxTTF, the sulfur, carbon and hydrogen atoms are colored in yellow, green and white, respectively. Adapted with permission from [18]. Copyright 2014 WILEY-VCH Verlag GmbH & Co. KGaA.

Considering the capability of the truxTTF electron-donor to host fullerenes promoted by the concave–convex complementarity (discussed in Section 3.1.2) [87], we expected that this electron-rich moiety was able to efficiently associate the hemifullerene C_30_H_12_ buckybowl in a similar fashion (i.e., in a bowl-in-bowl arrangement). To explore this possibility, an in silico investigation based on density functional theory (DFT) calculations was firstly carried out. Four different supramolecular truxTTF•C_30_H_12_ models (rationally constructed based on the crystallographic information on both C_30_H_12_ [108] and truxTTF [87]) were built up and fully optimized at the revPBE0-D3/cc-pVTZ level. Figure 16 displays the orientation of the four minimum-energy structures calculated for the truxTTF•C30H12 heterodimer. More structural details are given in Figure 17. In structures **A1** and **A2**, the convex surface of the C_30_H_12_ bowl perfectly matches the two concave cavities of the truxTTF host; that is, either through the cavity formed by the carbon backbone (structure **A1**) or through the cavity formed by the central benzene ring and the three dithiole rings (structure **A2**). Both structures can thus be seen as bowl-in-bowl arrangements where π–π interactions are maximized. The concave cavities of truxTTF and C_30_H_12_ can also interact, giving rise to heterodimers in which either a benzene or a dithiole ring of the truxTTF molecule is placed inside the concave cavity of the hemifullerene bowl (structures **A3** and **A4**, respectively). The optimized heterodimeric structures **A1**–**4** all show close intermolecular contacts in the 2.5–3.7 Å range, which is indicative of the stabilizing noncovalent interaction between both bowls. 

To evaluate the strength of the interaction between the truxTTF and C_30_H_12_ bowls, interaction energies of the previously-optimized heterodimers were computed at the same level of theory (revPBE0-D3/cc-pVTZ). The four supramolecular structures **A1**–**4** exhibit significant gas-phase interaction energies, ranging from −21.0 and −19.4 kcal/mol for **A1** and **A2**, respectively, to −25.2 and −28.5 kcal/mol for **A3** and **A4**, respectively. revPBE0-D3 calculations therefore predicted that the staggered structures are more stable than the bowl-in-bowl structures.

**Figure 18:**
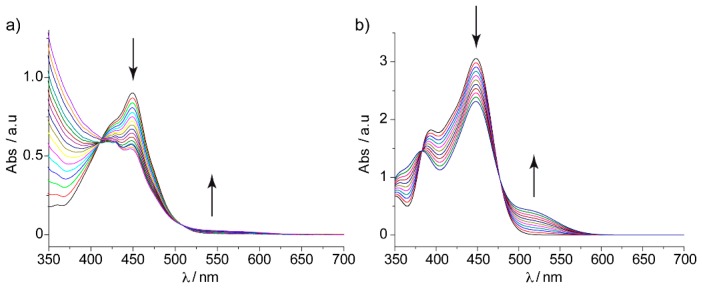
(**a**) Experimental UV–Vis spectra, as obtained during the titration of truxTTF (1.7 × 10^−4^ M) with C_30_H_12_ (0.8 × 10^−3^ M) in CHCl_3_ at room temperature; (**b**) TD-DFT simulation of the absorption spectrum of truxTTF as the ratio of truxTTF•C_30_H_12_ increases from 0 to 100% (B3LYP/cc-pVDZ calculations including CHCl_3_ as solvent for structure **A4**). Adapted with permission from [18]. Copyright 2014 WILEY-VCH Verlag GmbH & Co. KGaA.

Inspired by our theoretical findings, the N. Martín group performed a titration of truxTTF with C30H12 in dichloromethane at room temperature (Figure 18a). A decrease in the intensity of the truxTTF absorption at λ = 450 nm, accompanied by the increase of a broad band in the 500–600 nm region (Figure 18) was observed. From the UV–Vis experiments, an association constant of log Ka = 3.6 ± 0.3 for the truxTTF•C30H12 supramolecular complex in chloroform at room temperature was estimated.

To gain insight into the electronic nature of the absorption bands observed experimentally, and their evolution during the titration experiment, the lowest-lying singlet excited states (S_n_) of the truxTTF•C_30_H_12_ heterodimer and the constituting monomers were computed using the time-dependent DFT (TD-DFT) approach taking into account solvent effects. Only the results obtained for the most stable structure of truxTTF•C_30_H_12_ (**A4**) are discussed.

**Figure 19:**
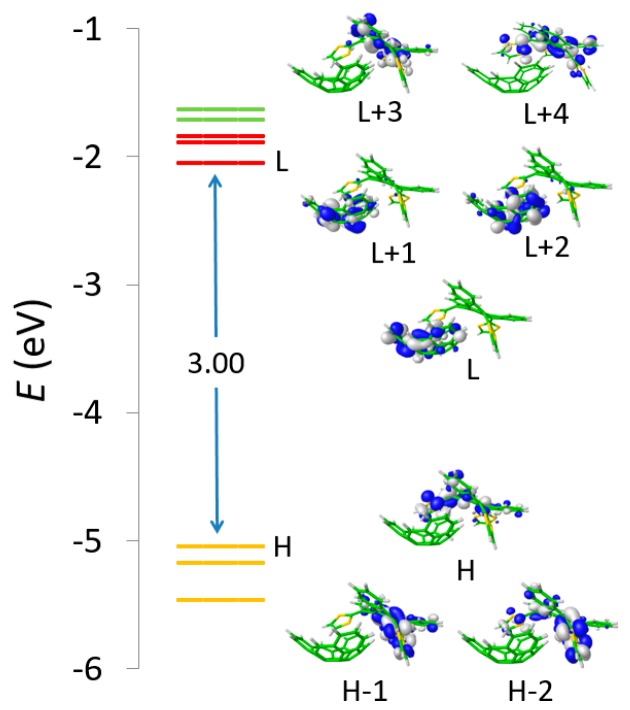
Isovalue contours (±0.03 a.u.) and energies calculated for the HOMOs and LUMOs of structure **A4** at the revPBE0-D3/cc-pVTZ level. H and L denote HOMO and LUMO, respectively. Adapted with permission from [18]. Copyright 2014 WILEY-VCH Verlag GmbH & Co. KGaA.

TD-DFT calculations predicted the first two excited states S_1_ and S_2_ at 537 nm (2.31 eV) and 516 nm (2.40 eV), respectively, above the ground state S_0_. The S_0_ → S_1_ and S_0_ → S_2_ electronic transitions have moderate oscillator strengths (ƒ) of 0.036 and 0.046, respectively, and were mainly described by one-electron promotions from the HOMO to the LUMO and LUMO+1, respectively. These transitions therefore imply a charge transfer from the electron-donor truxTTF, where the HOMO is located, to the electron-acceptor C_30_H_12_, where the LUMO and LUMO+1 spread (Figure 19), and are the major contribution to the band experimentally recorded in the 500–600 nm range. Calculations predicted several transitions (S_9_–S_11_) in the 450 nm region giving rise to the truxTTF-centered band originated from HOMO, HOMO−1 → LUMO+3, LUMO+4 one-electron excitations. A TD-DFT simulation of the experimental titration was performed by increasing the % of the truxTTF•C_30_H_12_ absorption spectrum with respect to that of the isolated truxTTF compound (see Figure 18b). The theoretical simulation was in sound agreement with the experimental evolution of the absorption spectrum, therefore supporting the formation of the supramolecular donor–acceptor truxTTF•C_30_H_12_ heterodimer and the appearance of a low-lying charge-transfer band in the region of 500–600 nm.

The formation of the charge-separated truxTTF^+^•C_30_H_12_^−^ species upon photoexcitation was further confirmed by femtosecond pump–probe experiments carried out by the group of Guldi. The time-evolution analysis of the spectroscopic data afforded rate constants of 6.6 × 10^11^ and 1.0 × 10^10^ s^−1^ for the charge separation and charge recombination dynamics, respectively.

This joint experimental and theoretical work demonstrated for the first time that a fullerene fragment mimics the charge-transfer behavior of the parent buckminster C_60_ buckyball in a donor–acceptor supramolecular assembly. From a theoretical point of view, counterintuitive staggered structures (**A3** and **A4**) were suggested as the most stable arrangements for the truxTTF•C_30_H_12_ supramolecular heterodimer. Remarkably, the supramolecular prediction at the DFT-D3 level was recently confirmed by high-level theoretical calculations at the DLPNO-CCSD(T) level of accuracy [109], and by another experimental–theoretical study conducted by us on related buckybowl systems (Section 3.2.2) [19]. The theoretical study of the truxTTF•C_30_H_12_ complex therefore represents a good example where quantum-chemical calculations were decisive to provide a deep insight into the supramolecular organization of truxTTF•C_30_H_12_, which turned out to be strikingly different compared to that found for its truxTTF•C_60_ homologue.

#### 3.2.2. Buckybowls for Donor–Acceptor Assemblies

**Figure 20:**
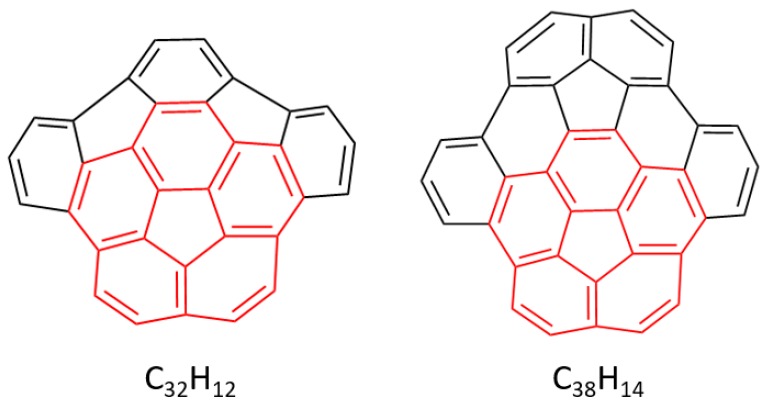
Chemical structure of corannulene-based C_32_H_12_ and C_38_H_14_ buckybowls. The corannulene skeleton is highlighted in red.

Encouraged by the study reported for the truxTTF•C_30_H_12_ complexation, we explored, by means of a combined experimental–theoretical investigation, the capability of similar carbon-based buckybowls of increasing size (Figure 20) to supramolecularly interact with the truxTTF electron-donor. In contrast to hemifullerene C30H12, the recently reported larger C32H12 and C38H14 buckybowls are corannulene-based fragments of C_60_ and C_70_ fullerene, respectively [110,111,112]. Such a difference in the aromatic core and the system size might be accompanied by fundamental differences in terms of electronic properties and/or supramolecular complexation.

**Figure 21:**
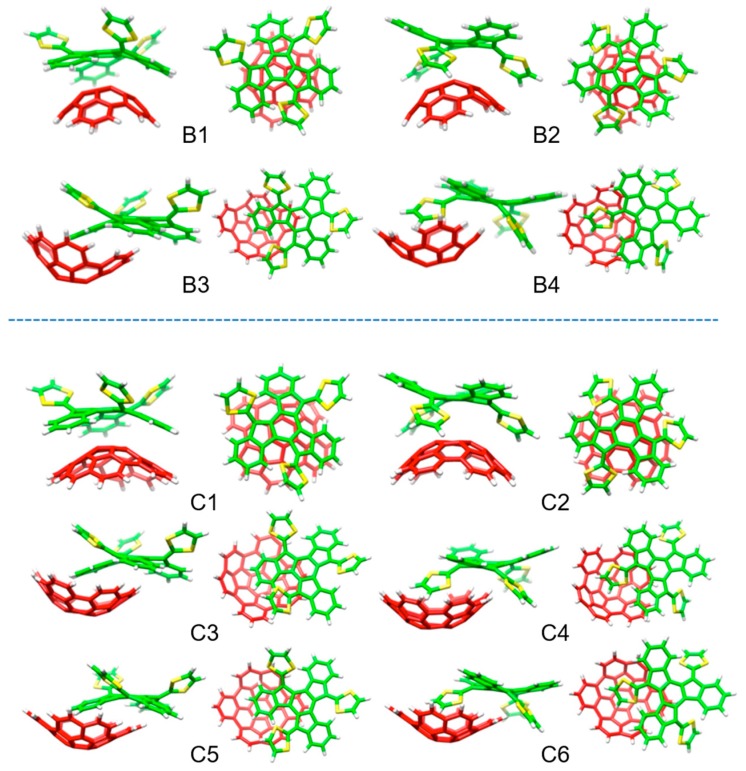
Minimum-energy structures and computed at the revPBE0-D3/cc-pVTZ level for the most stable conformations of the heterodimers formed by the C_32_H_12_ (**B1**–**4**) and C_38_H_14_ (**C1**–**6**) fullerene fragments with truxTTF (truxTT•C_32_H_12_ and truxTT•C_38_H_14_). Except for the carbon atoms of the C_32_H_12_ and C_38_H_14_ buckybowls depicted in red, the following color code is used: carbon in green, sulfur in yellow and hydrogen in white. Adapted with permission from Reference [19]. Copyright 2017 WILEY-VCH Verlag GmbH & Co. KGaA, Weinheim.

Based on our previous experience with hemifullerene C_30_H_12_ (Section 3.2.1), we expected that the larger C_32_H_12_ and C_38_H_14_ fragments were able to associate with truxTTF in a similar manner. To explore this hypothesis, we started by studying the supramolecular interaction in silico, by means of DFT-D3 calculations at the revPBE0-D3/cc-pVTZ level (Figure 21). 

In analogy to that previously obtained for C_30_H_12_, the corannulene-based C_32_H_12_ and C_38_H_14_ buckybowls may interact either through concave–convex bowl-in-bowl arrangements —structures **B1**–**2** for C_32_H_12_, and **C1**–**2** for C_38_H_14_— with a maximization of π–π interactions, or through a concave–concave staggered disposition, implying a mixture of π–π and CH···π noncovalent interactions —structures **B3**–**4** for C_32_H_12_, and **C3**–**6** for C_38_H_14_—.

Table 7 summarizes the binding energies calculated at the revPBE0-D3/cc-pVTZ level for the truxTT•C_32_H_12_ and truxTT•C_38_H_14_ complexes in comparison with those obtained for truxTT•C_30_H_12_. Briefly, the binding energy for the bowl-in-bowl structures was computed to be several kcal/mol less stable than the staggered dispositions in all cases. Among the latter, the arrangements in which the dithiole is placed inside the basin of the buckybowl fragment were always computed to be the most stable heterodimers ranging from −28.5 kcal/mol in C_30_H_12_, to −29.9 kcal/mol in C_32_H_12_ and to −34.2 kcal/mol in C_38_H_14_. These values suggested that the supramolecular interaction with the electron-donor truxTTF is reinforced upon increasing the buckybowl size due to the increasing number of weak NCIs originated from π–π and CH···π contacts.

In order to provide a more realistic description reflecting the strength of complexation at room temperature and in solution, the free energy of the dimerization process was theoretically estimated for all the possible conformers of truxTTF•C_32_H_12_ and truxTTF•C_38_H_14_, and compared with that computed for truxTTF•C_30_H_12_. Enthalpy and entropy corrections to the free energy were calculated at the B3LYP/cc-pVDZ level of theory. For the entropic part, the rigid-rotor harmonic-oscillator approximation (RRHO) was used as described by Grimme [79]. Solvent effects were included at the same level of theory using the Universal Solvation Model based on the Solute Electron Density (SMD) [113]. The reader is referred to [19] for further details.

Free energies in gas phase show that entropic effects are similar for both bowl-in-bowl and staggered dimers (compare *E*_int_ and Δ*G*_gas_ in Table 7). Upon inclusion of solvent effects (chloroform), the Δ*G*_theor_ values obtained indicate the same trends for the relative stabilities of the different supramolecular arrangements as predicted by the association energy (Table 7). Interestingly, only the staggered conformers provided negative values of Δ*G*_theor_, suggesting that bowl-in-bowl arrangements might not be formed in solution. For the three buckybowls, the staggered dimers in which the dithiole ring is placed inside the bowl basin were computed as the most stable structures, with Δ*G*_theor_ values of −4.29, −4.93 and −5.00 kcal/mol for truxTTF•C_32_H_12_, truxTTF•C_38_H_14_ and truxTTF•C_30_H_12_, respectively. Theoretical log *K*_a_ values were predicted in the range of 3–4, showing a perfect matching in the case of truxTTF•C_30_H_12_ (log *K*_a,theor_ = 3.7) with respect to the experimental value previously reported (log *K*_a_,_exp_ = 3.6 ± 0.3) [18]. 

With these promising theoretical findings in hand, the supramolecular association of truxTTF with the C_32_H_12_ and C_38_H_14_ buckybowls was experimentally corroborated by absorption titrations in several solvents at room temperature. Overall, the absorption features in the UV–Vis spectrum upon titration led to similar band evolution as that previously found in truxTTF•C30H12 (Figure 18). Multiwavelength analysis of the titration experiments allowed the estimation of association constants of log *K*_a_ = 2.9–3.3 for C_32_H_12_ and log *K*_a_ = 3.4–3.5 for C_38_H_14_, respectively. These experimental constants were in excellent agreement with the theoretical constants (Table 7), suggesting the formation of the staggered structures predicted theoretically as the preferred dispositions. It should be stressed that these staggered arrangements were finally confirmed by ^1^H-NMR experiments performed for the C_32_H_12_ and C_38_H_14_ heterodimers. In the case of C_32_H_12_, all the signals of C_32_H_12_ suffered slight and quantitatively similar upfield shifts upon complexation. Meanwhile, the signals corresponding to the truxene core of truxTTF appeared unaltered, and only the dithiole ring signals underwent upfield shifts of ca. 0.02 ppm (see [19] for further experimental details). The case of truxTTF•C_38_H_14_ was not straightforward and the ^1^H-NMR spectra pointed to a coexistence in solution of the staggered structures, with predominance of those in which the dithiole rings are inside the cavity of C_38_H_14_ (similarly to **B4** in Figure 21 for truxTTF•C32H12), again in perfect agreement with the DFT-D3 calculations (Table 7).

These experimental–theoretical outcomes constitute the first evidences that buckybowls, in contrast to fullerenes, are able to supramolecularly interact with electron-donor organic molecules in dispositions other than the typical concave–convex arrangements, maximizing both CH···π and π–π interactions. It is noteworthy that the structures implying the dithiole rings were found more stable than those only involving the carbon backbones, thus indicating the important role played by sulfur-mediated noncovalent interactions.

### 3.3. Carbon Nanotube Supramolecular Assemblies

Single-walled carbon nanotubes (SWNTs) are one of the most promising nanomaterials owing to their structural and electronic properties, and have attracted a lot of attention in the last years [114]. In general, chemical modifications are necessary to modulate and control their properties [115,116]. A particularly attractive strategy is to exploit NCIs to generate supramolecular architectures, since it guarantees the structural integrity of the nanotube and the strength of the interaction can be modulated by changing the structure of the host, its concentration, the solvent, and/or the temperature. In this regard, the quantification of the supramolecular interactions is of paramount relevance. However, there is no experimental standard method for the quantification of their supramolecular chemistry in solution/suspension. Recently, a simple procedure for the determination of association constants (*K*_a_) between soluble molecules and insoluble, heterogeneous carbon nanotube samples was proposed by some of us [117]. 

**Figure 22:**
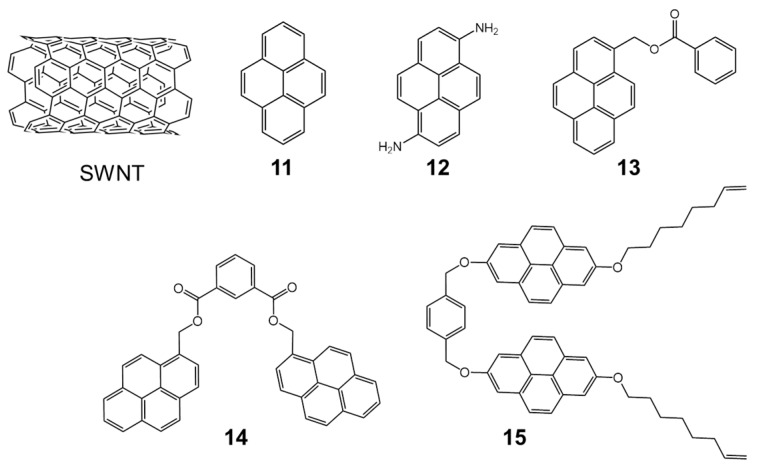
Chemical structure of a SWNT model and the hosts used to supramolecularly interact with SWNTs and test the experimental association constant protocol.

Figure 22 displays the chemical structures of the five host systems that were used to test the experimental method. Interestingly, the experimental methodology turned out to be sensitive to solvent effects, the size (diameter) of the SWNT and the hosts interacting with a particular SWNT. 

A thorough theoretical study by means of DFT-D3 calculations was carried out to corroborate and support the experimental findings. All supramolecular assemblies between hosts **11**–**15** and a SWNT model were optimized at the PBE0-D3/6-31G** level including the three-body dispersion correction (*E*^ABC^). The strength of the interaction between host and guest was calculated by means of two different quantities: *E*_int_ and *E*_bind_. Because of the small size of the double-zeta 6-31G** basis set employed, the basis set superposition error (BSSE) associated to the *E*_int_ values was corrected using to the counterpoise (CP) scheme.

**Figure 23:**
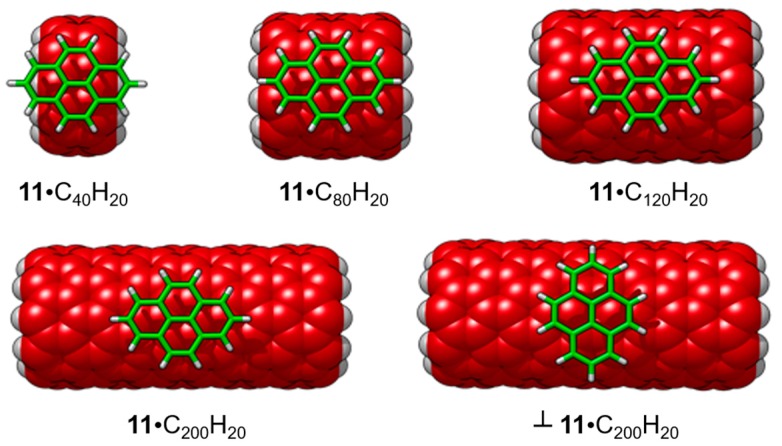
Minimum-energy geometries of parallel **11**•SWNT assemblies and the perpendicular **11**•C_200_H_20_ calculated at the PBE0-D3/6-31G** level from a semi-rigid optimization with fixed intramolecular parameters (see the original Reference [117] for further details). Carbon atoms of SWNTs are highlighted in red whereas the carbon atoms of pyrene are in green. Hydrogen atoms are depicted in white. Reproduced from Reference [117] with permission from the Royal Society of Chemistry.

Prior to analyzing the host-guest complexes, the effect of the length of the nanotube into the binding energy was assessed by increasing the SWNT size in the **11**•SWNT complex. The binding energy was shown to be nearly converged with SWNT sizes slightly larger than the host length (see Table 8 and Figure 23). A fragment of a zig-zag (10,0)-SWNT was used as a general model for the SWNTs in this regard.

**Figure 24:**
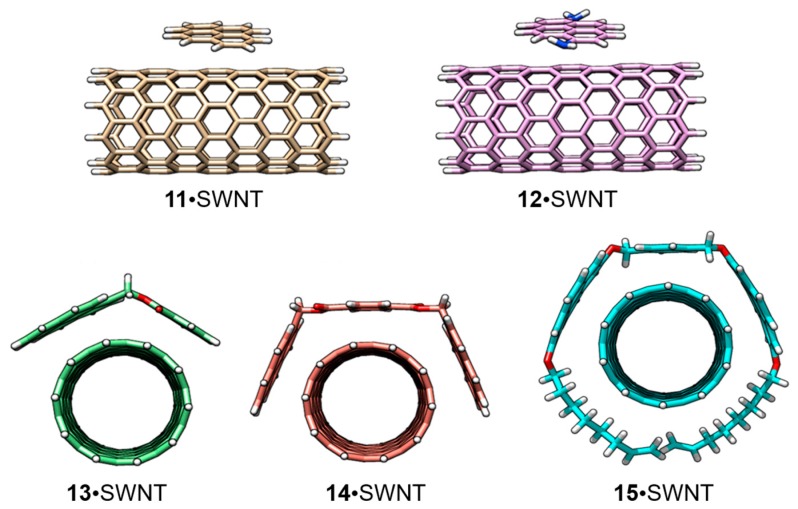
Minimum-energy geometry for the supramolecular assemblies formed by hosts **11**–**15** vs. SWNTs calculated at the PBE0-D3/6-31G** level of theory. Reproduced from [117] with permission from the Royal Society of Chemistry.

Figure 24 displays the minimum-energy geometries for the **11**–**15** hosts assembled with the SWNT model of C_160_H_20_ computed at the PBE0-D3/6-31G** level of theory in gas phase. Note that the size of the SWNT model (C_160_H_20_) was sufficiently large compared to all hosts studied and, therefore, the binding energy was not expected to vary in line with the previous length-dependence study for the **11**•SWNT complex (Table 8). Among the different closely energetic conformations of **11** over SWNT, the diagonal arrangement turned out to be the most stable, with close π–π contacts (3.2–3.5 Å). The interaction energy of **11**•SWNT was calculated to be −15.24 kcal/mol, which was slightly reduced to −14.84 kcal/mol for the binding energy as a consequence of the deformation energy penalty (0.59 kcal/mol). Moving from the pyrene system (**11**) to 1,6-diaminopyrene (**12**), additional *n*-π interactions resulting from close nitrogen···nanotube contacts emerge (ca*.* 4.0 Å). The *E*_int_ term of **12**•SWNT was found larger than that computed for **11**•SWNT by 1.3 kcal/mol, but this difference was not preserved in the binding energy (Table 9). The deformation energy, predicted to be 2.83 kcal/mol for **12**•SWNT, explained this trend. The incorporation of an extra aromatic ring in **13** gives rise to a significant increase of the interaction energy up to −23.68 kcal/mol, with close π–π benzene···SWNT (3.5 Å) and C=O···SWNT (3.2 Å) contacts. Bivalent tweezers-like hosts **14** and **15** were calculated to further enhance the supramolecular affinity towards SWNT, with *E*_int_ as large as −38.78 and −63.23 kcal/mol for **14**•SWNT and **15**•SWNT, respectively. The binding energy in complex **14**•SWNT (−36.42 kcal/mol) is indeed approximately the sum of *E*_int_ for its constituting moieties **11** and **13** (−14.84 + −21.52 = −36.36 kcal/mol), which supports the theoretical approach undertaken. Whereas the *E*_def_ of **14**•SWNT was calculated similar to **12**•SWNT and **13**•SWNT, it rose to 20.46 kcal/mol for **15**•SWNT owing to the optimal arrangement of the alkoxy chains around the nanotube (Figure 24). This disposition confers to **15**•SWNT an increased *E*_bind_ of −42.78 kcal/mol due to close CH···π contacts computed in the range of 2.7–3.2 Å, which contribute to the total binding energy approximately in 6 kcal/mol.

**Figure 25:**
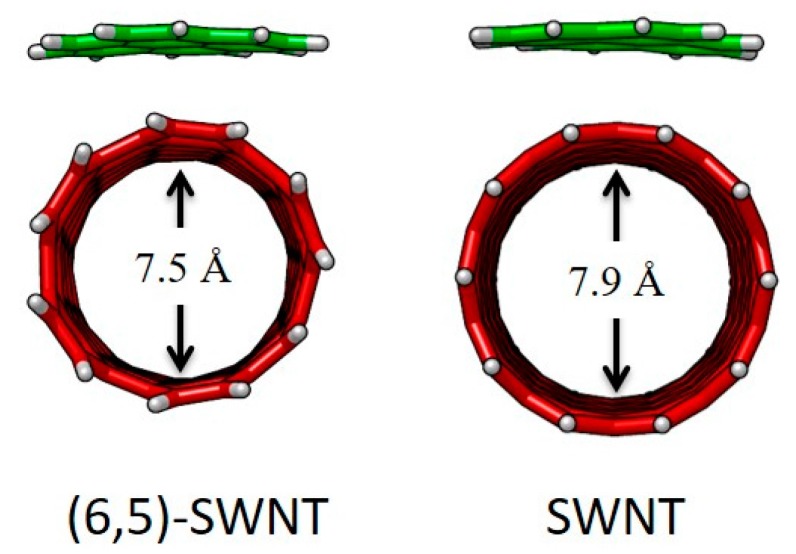
Side view of the supramolecular complex formed by **11** and two types of SWNTs. Carbon atoms of SWNTs are highlighted in red whereas the carbon atoms of pyrene are in green. Hydrogen atoms are depicted in white. Reproduced from [117] with permission from the Royal Society of Chemistry.

Finally, the influence of the structure of the nanotube in the stability of the host-guest assembly was assessed by comparing the binding energies of the SWNT model (C_160_H_20_) and (6,5)-SWNT (C_132_H_22_) with pyrene **11**. The *E*_int_ of **11**•(6,5)-SWNTs was found to be −13.85 kcal/mol, which is 1.4 kcal/mol smaller than the *E*_int_ of **11**•SWNTs. The minimum-energy optimized geometries calculated for the supramolecular complexes between pyrene and the two types of nanotubes (Figure 25) revealed subtle differences in terms of intermolecular contacts. The diameter of (6,5)-SWNT was calculated at 7.5 Å, slightly shorter than SWNT (7.9 Å), which causes a less efficient supramolecular assembly with pyrene. The deformation energy of **11**•(6,5)-SWNT was computed somewhat larger than that of **11**•SWNTs (Table 9), suggesting that the pyrene core was required to have a larger deformation to accommodate over the more-curved nanotube surface of (6,5)-SWNT. Moreover, the intermolecular contact area for **1**•(6,5)-SWNTs was estimated to be 0.5 Å^2^ smaller than **11**•SWNTs.

**Figure 26:**
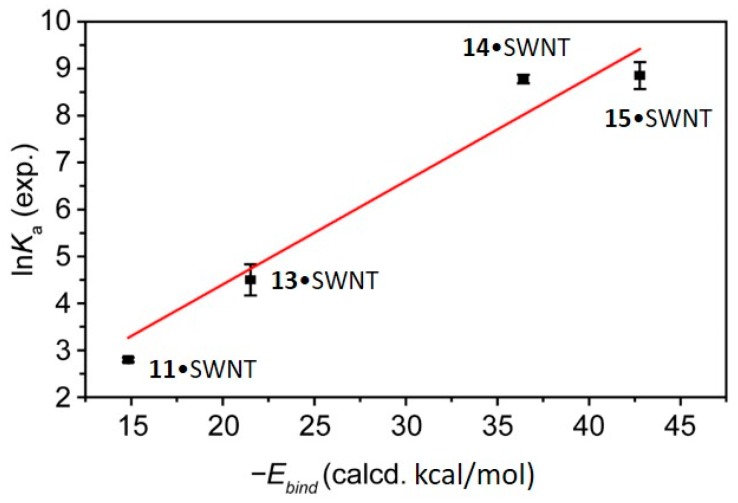
Plot of ln*K*_a_ vs. −*E*_bind_, comparing the experimental and calculated data.

Most remarkably, the calculated *E*_bind_ energies and the experimentally determined *K*_a_ values exhibited admirable quantitative agreement, despite the fact that solvent effects were not included in our calculations. A plot of the ln*K*_a_ vs. −*E*_bind_ for hosts **11**, **13**, **14** and **15** bound to SWNTs in THF at room temperature, the largest set for which comparable *K*_a_ data were obtained, is shown in Figure 26. Fixing the intercept to 0, the data fitted well (*r*^2^ = 0.984) to a straight line of slope 0.22 ± 0.01. Our analysis showed that the Δ*G*_bind_ determined experimentally was proportional to the calculated *E*_bind_.

This study was followed by an exhaustive combination of experiment, density functional theory (DFT) and molecular dynamics (MD) simulations for the quantitative analysis of the noncovalent interaction between (6,5)-SWNT, as host, and a set of pyrene derivatives with different electronic properties and surface area, as guests [118]. The supramolecular stabilization of the assemblies were explained in terms of dispersion forces as suggested by the linear correlation between the intermolecular contact area and the interaction energy, whereas electronic charge-transfer effects were ruled out. Molecular dynamics with explicit solvent molecules allowed us to predict binding constants in remarkable agreement with those derived from experiment, and to disentangle the molecular details underlying the adsorption process from low to high SWNT surface coverage. For further details, the reader is referred to the original work [118]. 

### 3.4. exTTF-Graphene Supramolecular Assembly

As already mentioned in Section 3.1, the concave geometry of the exTTF electron-donor can be exploited to supramolecularly recognize fullerenes by means of efficient π–π interactions owing to the concave–convex complementarity. Nevertheless, there are numerous examples where planar π-conjugated platforms (particularly porphyrins) are able to noncovalently interact with fullerene and nanotubes despite the planar–convex mismatch [80,119]. Inspired by this fact, we explored the possibility of exTTF to establish stabilizing noncovalent interactions with planar graphene, regardless of its biconcave geometry. Experimentally, the positive interaction between exTTF and graphene was studied by means of a multivalent approach, where gold nanoparticles were decorated with exTTF units (exTTFAuNPs) [120]. The interaction between the exTTFAuNPs with graphene was confirmed by a series of spectroscopic (UV–Vis and Raman) and transition electron microscopy (TEM) techniques.

**Figure 27:**
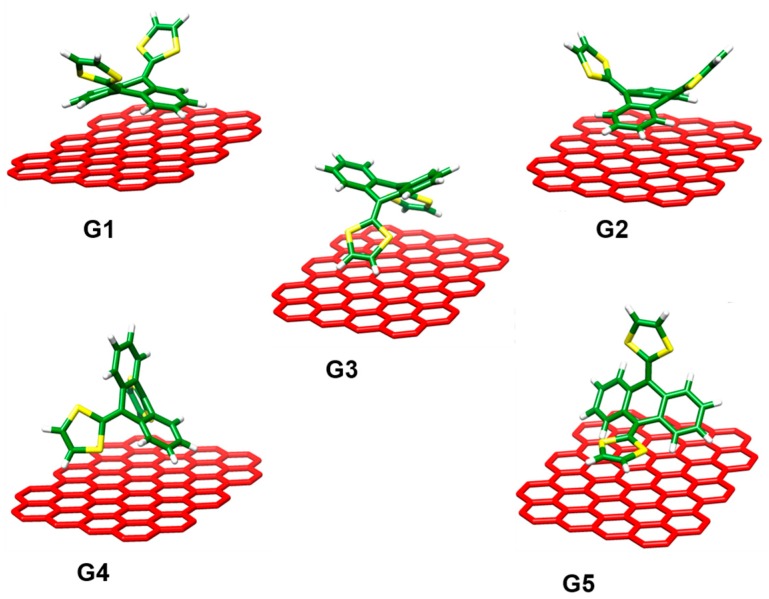
Minimum-energy structures computed for the exTTF-graphene models (**G1**–**G5**) at the revPBE0-D3/cc-pVDZ level. Carbon atoms of exTTF are depicted in green, sulfur in yellow and hydrogen in white. Carbon atoms of the graphene sheet are depicted in red and hydrogen atoms have been omitted for clarity. Adapted with permission from [120]. Copyright 2013 WILEY-VCH Verlag GmbH & Co. KGaA.

Notwithstanding the precise experimental information obtained, several fundamental questions remained unsolved. For instance, how can the biconcave geometry of the exTTF compound be able to efficiently interact with the graphene sheet? To answer this question, and gain insight into the supramolecular recognition process between exTTF and graphene, we performed revPBE0-D3 calculations on an exTTF-graphene model. The exTTF unit was placed in different orientations over a polyaromatic hydrocarbon (PAH) graphene-like molecule including 31 benzene rings (C_84_H_24_). Figure 27 displays the minimum-energy structures (**G1**–**G5**) calculated for the exTTF-graphene supramolecular complex at the revPBE0-D3/cc-pVDZ level. In structures **G1** and **G2**, the interaction of the exTTF fragment with graphene mainly occurs through the concave anthracene backbone, whereas in structure **G3** the interaction takes place through the concave dithiole face. Structures **G1** and **G2** are mainly governed by π–π interactions, whereas structure **G3** involves close CH···π interactions (~2.7 Å) between the terminal hydrogen atoms of the dithiole rings and the graphene sheet. The exTTF molecule can also interact with the graphene sheet through a benzene ring and the sulfur atoms (structure **G4**), or through a dithiole ring (structure **G5**). 

Structures **G4** and **G5** imply a mixture of π–π and CH···π interactions. All optimized structures **G1**–**G5** showed close intermolecular distances in the 3.1–4.0 Å range, which are the structural signature of stabilizing noncovalent interactions. Remarkably, despite the electron-donor character of exTTF, our calculations predicted negligible contributions from charge-transfer interactions (<0.005e). 

**Figure 28:**
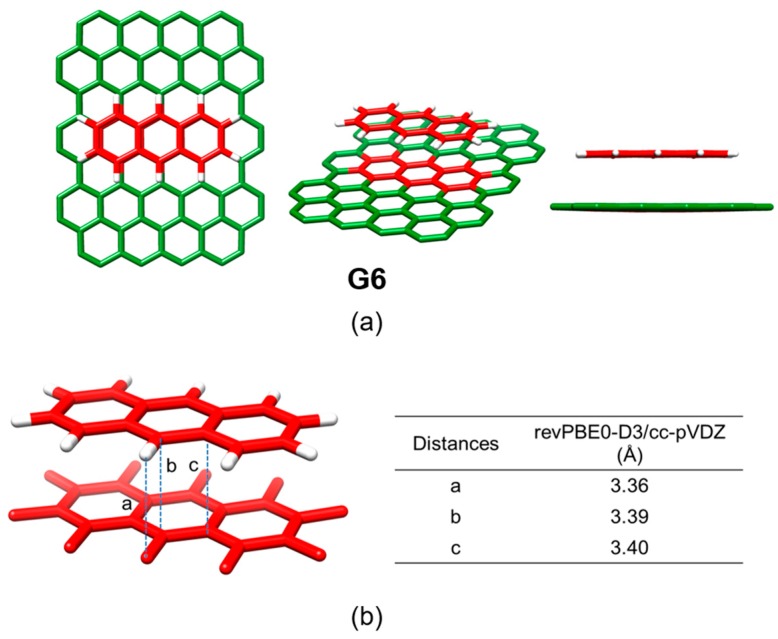
(**a**) Top and side views of the minimum-energy geometry computed for anthracene-graphene (**G6**) at the revPBE0-D3/cc-pVDZ level. The intermolecular interacting regions of anthracene and graphene are colored in red; (**b**) Magnification of the interacting region emphasizing the most representative intermolecular distances collected in the table. Adapted with permission from [120]. Copyright 2013 WILEY-VCH Verlag GmbH & Co. KGaA.

To estimate the interaction energies of the exTTF-graphene models **G1**–**G5**, single-point energy calculations were performed on the previously-optimized structures using the revPBE0-D3 functional, and the more extended triple-ζ cc-pVTZ basis set. Structures **G4** and **G5** were found to be the most stable structures with interaction energies of −22.2 and −25.2 kcal/mol, respectively. Significant *E*_int_ of −19.0, −20.7 and −16.8 kcal/mol were also calculated for structures **G1**–**G3**. These theoretical outcomes clearly confirmed the presence of a significant stabilizing interaction between the exTTF derivative and a graphene sheet. However, the influence of the nonplanar geometry of exTTF on the supramolecular association with graphene was still unclear. To explore this effect, the interaction of the graphene sheet with a planar π-conjugated motif like anthracene (the central backbone of exTTF) was also computed in an eclipsed-like disposition similar to structure **G6** (Figure 28). The interaction energy computed at the revPBE0-D3/cc-pVTZ level for this anthracene–graphene model was −20.4 kcal/mol, which is higher than that computed for its homologous structure **G1** but significantly smaller than those computed for structures **G4** and **G5**. The remarkable increase of the interaction energies for structure **G4** and **G5** with respect to the anthracene–graphene model clearly reveals that the planarity of the π-conjugated motif does not necessarily favor a stronger supramolecular association with a graphene sheet. For instance, despite the concave–planar mismatch, the exTTF system can orientate in different ways to interact strongly with a graphene sheet. These final theoretical findings allowed us to confirm, counterintuitively a priori, that planarity is not a *sine qua non* condition for a structure to bind effectively graphene.

Recently, a computational approximation based on molecular mechanics/molecular dynamics (MM/MD) demonstrated to be particularly useful for characterizing the supramolecular organization of C_60_-based molecules endowed with one or three pyrene units when interacting with graphene to form mono- and tripodal graphene nanobuds [121]. This is another example that clearly highlights the potential of computational molecular modelling (classical or quantum) in the field of supramolecular chemistry. 

## 4. Conclusions

Carbon nanoforms are at the forefront in current science owing to their potential application in a variety of fields. The supramolecular chemistry of these carbon-based nanoforms is of wide interest since the functionalization with π-conjugated compounds by means of noncovalent interactions allows maintaining the integrity of the nanoforms while modulating some properties such as the solubility or stability of the resulting assemblies.

In this review, we have shown how quantum-chemical calculations can be used as a powerful characterization tool to gain deep insights into the self-organization of different carbon-based nanoforms (such as fullerenes, fullerenes fragments, carbon nanotubes and graphene) with different electron-rich π-conjugated motifs in the generation of appealing supramolecular complexes governed by noncovalent interactions.

With respect to fullerene-based supramolecular complexes, quantum-chemical calculations have been fundamental to: (i) unveil the nature of the stabilizing noncovalent interactions (π–π, *n*–π and CH···π) that govern the supramolecular assembly formed by extended tetrathiafulvalene (exTTF), decorated with crown-ether groups of increasing size, and fullerene C_60_; (ii) provide an in-depth structural characterization concerning the supramolecular organization between the electron-donor truxene-tetrathiafulvalene (truxTTF) and the electron-acceptor C_60_; (iii) thoroughly disentangle the role of the different interactions (dispersion, exchange, electrostatic, induction) that contribute to the stabilization of the porphyrin–C_60_ assemblies; and finally; (iv) explain the negative cooperative effects as a consequence of the depletion of electron density the electron density in ditopic porphyrin receptors upon insertion of the first fullerene molecule.

In the buckybowl-based heterodimers formed with the electron-donor truxTTF, theoretical calculations were decisive to propose the staggered arrangements as most stable supramolecular structures, which seemed to be a priori very counterintuitive but were later confirmed by ^1^H-NMR experiments. In the example of single-walled carbon nanotubes (SWCNs) assemblies, the quantum-chemical calculations were helpful to support the experimental protocol to estimate association constants between soluble molecules and insoluble, heterogeneous carbon nanotube samples, which is crucial to understand the supramolecular chemistry of these SWCN compounds for current technological applications. Finally, theoretical calculations predicted a stabilizing interaction between graphene and the electron-donor exTTF despite the geometrical planar–curved mismatch. Remarkably, the theoretical outcomes demonstrated that planarity is not a prerequisite for a supramolecular recognition motif to effectively interact with graphene.

Despite the current success in the development of feasible quantum-chemical methods able to accurately describe relatively large-size supramolecular complexes, the molecular size is still critical for routine quantum-chemical calculations on molecular systems of more than 1000 atoms. Notwithstanding, new avenues of research concerning the development of large-scale quantum-chemical techniques allow dealing with very large molecular systems [64,122,123,124], and point to a bright future for the field of quantum chemistry and molecular modelling applied to supramolecular chemistry problems.

## Figures and Tables

**Table 1:** Composition of the exchange-correlation density functionals used along this review.

Functional	Type ^1^	*E_x_*[*ρ*]	*E_c_*[*ρ*]	$w_{HF}$	Reference
revPBE0-D3	H-GGA	revPBE	revPBE	0.25	[48,49]
PBE0-D3	H-GGA	PBE	PBE	0.25	[48,50]
B3LYP	H-GGA	B88	LYP, VWN	0.20	[51,52]
MPWB1K	H-M-GGA	mPW	B95	0.44	[53,54,55]
B97-D/D3	GGA	B97	B97	0.00	[56,57]

^1^ H-GGA means a hybrid density functional based on the generalized gradient approximation (GGA). H-M-GGA denotes a hybrid meta-GGA functional.

**Table 2:**
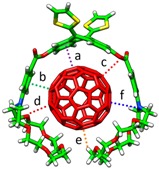
Intermolecular distances (**a**–**f**, in Å) and binding energies (*E*_bind_, in kcal/mol) calculated at the B97-D/cc-pVDZ and revPBE0-D3/cc-pVTZ levels, respectively, for the exTTF•C_60_ and **1**–**6**•C_60_ complexes [84] ^1^.

Complex	a	b	c	d	e	f	Ebind (kcal/mol)
exTTF•C_60_	3.42	-	-	-	-	-	−10.24
1•C_60_	3.46	2.99	3.30	3.19	2.61	-	−39.69
2•C_60_	3.45	2.95	3.42	2.79	2.69	-	−44.76
3•C_60_^2^	3.49 ^3^	2.98 ^3^	3.44 ^3^	2.85	2.50	-	−54.36
4•C_60_	3.37	3.41	3.25	3.56	2.57	4.14	−36.77
5•C_60_	3.37	3.06	3.14	3.14	2.54	3.50	−43.33
6•C_60_	3.45	3.25	3.16	-	-	-	−22.85

^1^ **a** is the distance between the centroid of the lateral benzene rings of exTTF and that of the closest benzene rings of C_60_; **b** is the distance between the centroid of the benzene ring of the benzoate moiety and the center of the closest C_60_ 6:6 double bond; **c** is the distance between the benzoate *sp*^3^ oxygen and the closest carbon atom of C_60_; **d** and **e** are the shortest O···C_60_ and H···C_60_ distances, respectively, between the crown ether and C_60_; **f** is the distance between the nitrogen atom of the aza-crown ether and the closest carbon atom of C_60_. ^2^ Two additional π–π interactions between the outer benzene rings of the crown ethers and C_60_ were computed at 3.13 and 3.68 Å. ^3^ Average values.

**Table 3:** DFT-optimized (B97-D/6-31G*) intermolecular distances (a–d, in Å) characterizing the **8-M**•**7** associates, and binding energies (*E*_bind_, in kcal/mol) computed at the PBE0-D3/cc-pVTZ level [92] ^1^.

Complex	M–C_60_ (a)	NH···O (b)	CH···C_60_ (c)	CH···C_60_ (d)	*E*_bind_
8-2H•7	2.756	1.848	2.679	2.577	−92.4
8-Co.•7	2.119	1.850	2.642	2.573	−93.8
8-Ni•7	2.793	1.842	2.623	2.610	−88.7
8-Cu•7	2.754	1.846	2.662	2.599	−91.3
8-Zn•7	2.701	1.845	2.689	2.591	−92.8

^1^ See Figure 10 for the definition of the intermolecular distances a–d. For further details, the reader is referred to the original work ([92]).

**Table 4:** Binding energy (kcal/mol), metal–ethylene distance *d* (Å), and natural population analysis (NPA) charge of the porphyrin central atom (M = 2H. Co., Ni, Cu, Zn) calculated at the PBE0-D3/cc-pVTZ level of theory for the simplified porphyrin–ethylene associates [92].

Complex	*E*_bind_	*d*	M Charge
2HP•C_2_H_4_	−4.636	2.986	+0.942
CoP•C_2_H_4_	−8.534	2.619	+0.720
NiP•C_2_H_4_	−4.530	3.177	+0.733
CuP•C_2_H_4_	−5.965	2.997	+1.006
ZnP•C_2_H_4_	−8.047	2.749	+1.223

**Table 5:** Energy decomposition (in kcal/mol) calculated at the SAPT0/def2-TZVP level for closed-shell porphyrin–ethylene systems with M = 2H, Ni, Zn [92].

Interactions	2HP•C_2_H_4_	NiP•C_2_H_4_	ZnP•C_2_H_4_
electrostatic	−2.794	−5.277	−16.212
exchange	7.046	8.605	22.101
induction	−0.690	−0.728	−3.900
dispersion	−7.033	−7.055	−10.521
TOTAL	−3.472	−4.455	−8.532

**Table 6:** Binding energies computed at the B97-D3/(cc-pVTZ+LANL2DZ) level for the host–guest supramolecular associates with stoichiometry 1:1 and 1:2 [90].

Complex	*E*_bind_ (kcal/mol)
**9•7**	−108.19
**9•7_2_**	−211.05
**10•7**	−98.40
**10•7_2_-*anti***	−195.40
**10•7_2_-*syn***	−200.20

**Table 7:** Thermodynamic parameters (in kcal/mol) including binding energy (*E*_bind_), free energy in gas phase (Δ*G*_gas_), and free energy including solvent effects (Δ*G*_theor_) for the dimerization process. Theoretical and experimental log *K*_a_ values are also included [19] ^1^.

Heterodimer		*E*_bind_	Δ*G*_gas_	Δ*G*_theor_	log *K*_a,theor_	log *K*_a,exp_
truxTTF•C_30_H_12_	A1	−21.02	−8.27	1.13	3.7	3.6
	A2	−19.38	−5.98	2.64		
	A3	−25.23	−10.88	−2.96		
	A4	−28.52	−14.34	−5.00		
truxTTF•C_32_H_12_	B1	−20.44	−3.87	5.07	3.2	2.9–3.3
	B2	−19.97	−4.24	3.67		
	B3	−24.69	−6.95	0.97		
	B4	−29.91	−12.84	−4.29		
truxTTF•C_38_H_14_	C1	−23.37	−4.32	5.81	3.6	3.4–3.5
	C2	−21.63	−3.46	4.80		
	C3	−29.09	−9.72	0.51		
	C4	−33.48	−14.39	−3.75		
	C5	−31.57	−11.71	−2.65		
	C6	−34.24	−14.94	−4.93		

^1^ The reader is referred to the original work [19] for further computational details and experimental specifications; ^2^ **C5** and **C6** conformers are analogous to **C3** and **C4**, respectively, in which the buckybowl is rotated by ~90° with respect to the truxTTF.

**Table 8:** Binding energy depending on the nanotube length for the parallel and perpendicular dispositions of the supramolecular **11**•SWNT complex calculated at the PBE0-D3/6-31G** level of theory [117].

		*E*_bind_ (kcal/mol)	
Disposition	SWNT Length	Semi-Rigid	Fully Relaxed
Parallel	11•C_40_H_20_	−11.05	−11.25
	11•C_80_H_20_	−17.80	−18.11
	11•C_120_H_20_	−21.79	−21.76
	11•C_200_H_20_	−21.18	−21.42
Perpendicular	11•C_40_H_20_	−10.88	−11.12
	11•C_80_H_20_	−16.40	−17.11
	11•C_120_H_20_	−19.46	−19.60
	11•C_200_H_20_	−18.85	−17.97

**Table 9:** Energy parameters (kcal/mol) and intermolecular contact area (CA, in Å^2^) calculated for the interaction between hosts **11**–**15** and SWNT guests at the CP-corrected PBE0-D3/6-31G**+*E*^ABC^ level [117].

System	*E*_int_	*E*_def_	*E*_bind_	CA ^1^
11•(6,5)-SWNTs	−13.85	0.81	−13.04	42.20
11•SWNTs	−15.24	0.59	−14.84	42.70
12•SWNTs	−16.53	2.83	−13.70	47.25
13•SWNTs	−23.68	2.16	−21.52	75.30
14•SWNTs	−38.78	2.36	−36.42	126.85
15•SWNTs	−63.23	20.46	−42.78	188.55

^1^ The intermolecular contact area (CA) was calculated using the UCSF Chimera 1.7 software according to the formula: (area of the host + area of the guest − area of the complex)/2, where the area refers to solvent-excluded molecular surfaces, composed of probe contact, toroidal, and reentrant surface.
